# Transient High-Harmonic Spectroscopy in an Inorganic–Organic Lead Halide Perovskite

**DOI:** 10.1021/acs.jpclett.3c02588

**Published:** 2023-11-28

**Authors:** Maarten L. S. van der Geest, Jeroen J. de Boer, Kevin Murzyn, Peter Jürgens, Bruno Ehrler, Peter M. Kraus

**Affiliations:** † Advanced Research Center for Nanolithography (ARCNL), Science Park 106, 1098 XG Amsterdam, The Netherlands; ‡ Center for Nanophotonics, AMOLF, Science Park 102, 1098 XG Amsterdam, The Netherlands; ¶ Max-Born-Institute for Nonlinear Optics and Short Pulse Spectroscopy, D-12 489 Berlin, Germany; § Department of Physics and Astronomy, and LaserLaB, Vrije Universiteit, De Boelelaan 1105, 1081 HV Amsterdam, The Netherlands

## Abstract

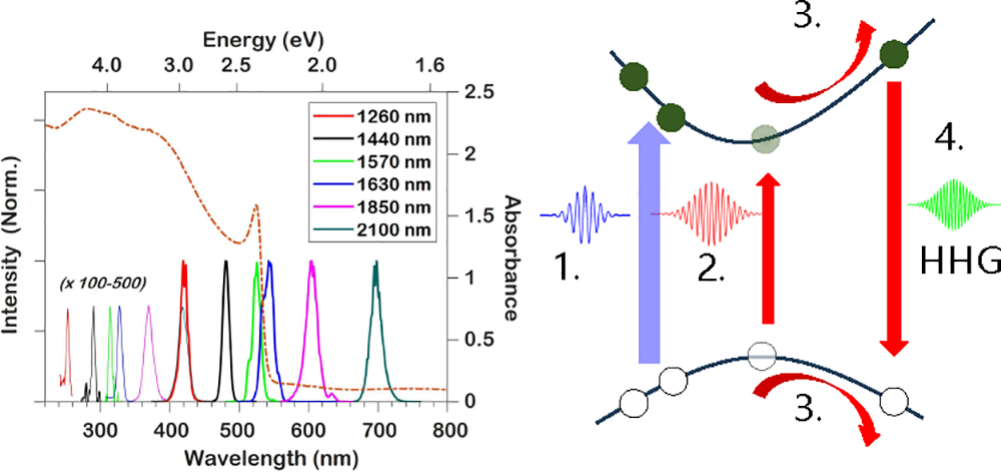

High-harmonic generation is the frequency upconversion of an intense femtosecond infrared laser in a material. In condensed-phase high-harmonic generation, laser-driven currents of coherently excited charge carriers map the electronic structure onto the emitted light. This promises a thus far scarcely explored potential of condensed-phase time-resolved high-harmonic spectroscopy for probing carrier dynamics. Here, we realize this potential and use time-resolved solid-state high-harmonic spectroscopy from a laser-excited methylammonium lead bromide (MAPbBr_3_) thin film, a key material in perovskite solar cells, for measuring carrier cooling and relaxation on femto- and picosecond time scales. Through comparison with transient absorption, we show the links between carrier dynamics and experimental observables of generated harmonics. By highlighting and understanding the interplay of these dynamics, we demonstrate transient optical control over the emission of solid-state high-harmonic generation in MAPbBr_3_.

Lead halide perovskites (APbX_3_ with A = methylammonium (MA, CH_3_NH_3_), formamidinium, Cs and X = Cl, Br, I) are promising materials for optoelectronic devices, such as light-emitting diodes, photovoltaics, and radiation detectors.^1−5^ This is due to their tunable bandgap, remarkable charge transport capabilities, high photoluminescence (PL) quantum efficiencies, power conversion efficiencies, and facile solution-based processing.^5−9^ Nonlinear optical responses in APbX_3_ perovskites at high laser intensities have been extensively investigated, including the optical Kerr effect, multiphoton absorption (MPA), and solid high-harmonic generation (SHHG).^10−14^ Ultrafast electron-dynamics are of great importance for the aforementioned applications, especially regarding nonlinear optical characteristics. These dynamics, such as recombination time and dominant recombination process, are strongly affected by the carrier density as demonstrated in transient absorption (TA) experiments.^15−19^

SHHG is a nonlinear process in which photons of a fundamental wavelength are upconverted to a harmonic multiple using a solid as generating medium.^20^ The SHHG behavior is material-, (micro)structure-, wavelength-, and fluence-dependent.^11,21−23^ Utilizing its ultrafast nature and sensitivity to (transient) electronic band structure, SHHG was recently demonstrated to be able to probe electronic phase-transitions.^24^ Additionally, recent experiments have demonstrated optical control over the harmonic generation process in various solid-state materials ranging from atomically thin 2D materials to bulk crystals.^25,26^ Probing gas-phase photochemistry via time-resolved high-harmonic spectroscopy from molecular gases has successfully been done in a plethora of systems.^27^ One particular strength of gas-phase high-harmonic spectroscopy is the extreme sensitivity of HHG to small excitation fractions due to the homodyne advantage: Unexcited molecules act as local oscillators against which the dynamics of excited molecules are measured by interference.^28−30^ This all motivates using SHHG to measure dynamics in complex materials.

In this work, we use SHHG as a tool for investigating the ultrafast dynamics of hot carrier cooling (HCC) and recombination after single-photon excitation (i.e., we overcome the band gap with one photon) and provide new insights into coherent control of SHHG. By systematically comparing our SHHG measurements at multiple near-infrared (NIR) driving wavelengths at a fixed pump fluence to a transient absorption (TA) measurement and simulated electron impact ionization (EII), we link the measured transient SHHG signal changes and frequency shifts to known excitation, cooling, and recombination processes in methylammonium lead bromide (MAPbBr_3_). Our results reveal how photophysical primary processes cause amplitude reduction and center frequency shifts in SHHG, and vice versa introduce SHHG as powerful probing mechanisms for following carrier dynamics in organic thin-film materials. Combined with the previous insight that HHG is driven by laser-induced currents in bands, which led to all-optical band-structure reconstruction,^31^ our results pave the way for time-dependent band structure measurements. This capability is particularly relevant for materials where excitation induces drastic changes in the band structure, such as phase transitions in strongly correlated materials.

Sample fabrication, characterization, and experimental details can be found in the Supporting Information. The fabrication process yields a high-quality, 300 nm thick, uniform, polycrystalline MAPbBr_3_ film with grain sizes between 500 nm and 8 μm on a fused silica substrate. The SHHG signal, as a function of peak intensity, was measured in transmission. We explored the SHHG-intensity dependence of the third, fifth, and seventh harmonic orders (HOs) at NIR driving wavelengths (λ_0_) between 1260 and 2100 nm, with examples of third and fifth harmonic spectra depicted in Figure 1A. The ultraviolet–visible (UV–vis) absorption spectrum of the MAPbBr_3_ sample under normal incidence is depicted in Figure 1A, showing the characteristic exciton absorption feature at 526 nm.^32^ Tuning the driving wavelength such that the third harmonic is generated below the bandgap yields a very close adherence to a perturbative *I*^3^ power-law dependence which starts to deviate and saturate at higher intensities, as can be seen in Figure 1B, possibly due to photophysical damage from the NIR field. Furthermore, it was found that the deviation of the 5HG and 7HG harmonic yield from perturbative *I*^5^ and *I*^7^ power law dependence, respectively, occurs at lower intensities than for the third harmonic, attributable to absorbances higher than 1.5 (Figure 1A) over the entire film thickness at the respective wavelengths of all fifth and seventh harmonics depicted in Figure 1B–D. We observed that THG driven at 1630 and 1570 nm, making it resonant with the bandgap (530 nm), is more efficient than THG either above or below bandgap, due to favorable resonance effects, while simultaneously avoiding excessive reabsorption below 500 nm. For driving wavelengths where the THG center wavelength is shorter than the bandgap (Figure 1D), saturation occurred at lower fluences, but adherence to an *I*^3^ power law for the third harmonic was still noticeable up to the damage threshold. This furthermore extends earlier reported intensity-dependence data for THG at λ_0_ = 1260 nm.^33^ The fifth harmonics in Figure 1D display a weaker intensity dependence, likely again due to the significant reabsorption. Saturation or reduction in harmonic intensity is observed above 90 GW/cm^2^, possibly signifying an intensity above which photobleaching occurs. Because of the added pump pulse, the experimental driving intensity was thus fixed to 50 GW/cm^2^ for all NIR driving wavelengths in the pump–probe experiments, which will be discussed below.

**Figure 1:**
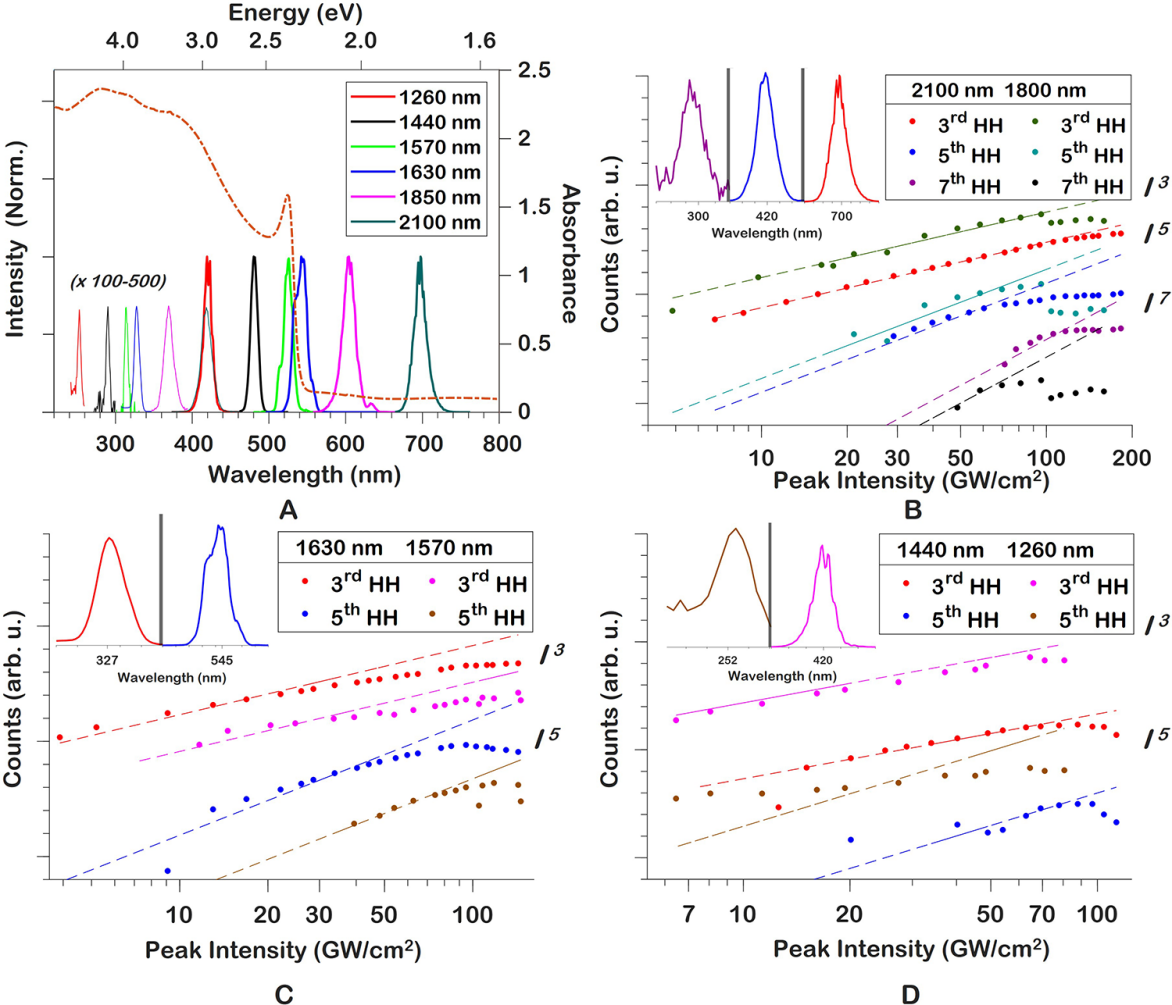
(A) UV–vis absorption spectrum of the MAPbBr_3_ thin-film sample under normal incidence (red, right *y*-axis) overlaid with harmonic spectra of six driving wavelengths normalized to the third harmonic intensity. Fifth harmonics have been scaled for clarity. (B–D) Peak intensity dependence of third harmonic generation (THG) and fifth harmonic generation (5HG) of six infrared driving wavelengths as well as (B) seventh harmonic generation (7HG) of λ_0_ = 1800 and 2100 nm. Insets of panels B–D depict normalized harmonics of λ_0_ = 2100, 1630, and 1260 nm, respectively. Curves were offset for clarity.

For the pump–probe experiments, the 400 nm pump fluence was fixed at ∼0.3 mJ/cm^2^ to photoexcite the sample by single-photon excitation. This fluence yields a carrier density of (2.7 ± 1.1) × 10^19^ cm^–3^, corresponding to a 0.4–0.8% excited-state fraction, based on unit cells per cm^3^. Further experimental details can be found in the Supporting Information. To understand the photophysical primary processes, we first measure a time-resolved transient absorption spectrum that we systematically compare to the transient SHHG results. The relatively high carrier density results in large changes in the TA spectrum as a function of pump–probe delay, depicted in Figure 2A. The TA measurements were conducted on a separate, purpose-built setup with experimental details provided in the Supporting Information. The large decrease in absorbance around 2.3 eV corresponds to the ground-state bleach (GSB).^17^ The GSB is exceptionally broad in this TA measurement due to the high carrier density. The broadening of the GSB is attributed to the Burstein–Moss (BM) effect, where states close to the conduction-band edge are populated, while the highest VB states are depleted, causing the effective bandgap to become larger. This effect can dominate over bandgap renormalization (which typically reduces the bandgap) at high intensities as present in our work.^34,35^ Using an estimate based on the BM shift of the GSB in Figure 2B (+1.4 ps, red line), we find a carrier density of (2.1 ± 1.1)× 10^19^ cm^–3^ for the presented TA results. This matches well with the estimated excitation fluence used in the TA measurement.^36^Figure 2C is produced by spectrally averaging the transient absorbance around the GSB between 2.31 and 2.48 eV. We fit the GSB to exponentials of the form *F*(τ) = ∑_*i*=0_^3^α_*i*_ exp(−τ/τ_*i*,GSB_) (Figure 2C), where α_*i*_ can be positive or negative to capture a decay or rise, respectively. The first part of the GSB around the pump–probe overlap (time-zero, *t*_0_) is obscured by the 0.2 ps instrument-response function (IRF, τ_0,GSB_) but has a slower component with a time constant of τ_1,GSB_ = (0.53 ± 0.05) ps. This fast process is associated with HCC in which the initial photoexcited nonequilibrium distribution cools toward a Fermi–Dirac distribution through various scattering mechanisms.^35^ The subsequent recovery of the GSB is found to consist of two components of τ_2,GSB_ = (5.2 ± 0.3) ps and τ_3,GSB_ > 100 ps. These correspond to bimolecular and unimolecular recombination processes, respectively.^16^ The observed decay constants are the weighted averages of all bimolecular and unimolecular processes that occur, such as photoluminescence and thermal relaxation. A large errorbar is associated with τ_3,GSB_, since its value cannot be reliably fitted. Time constants reported in the literature on the unimolecular recombination rate constant start at 100 ps up to tens of nanoseconds.^16,37^

**Figure 2:**
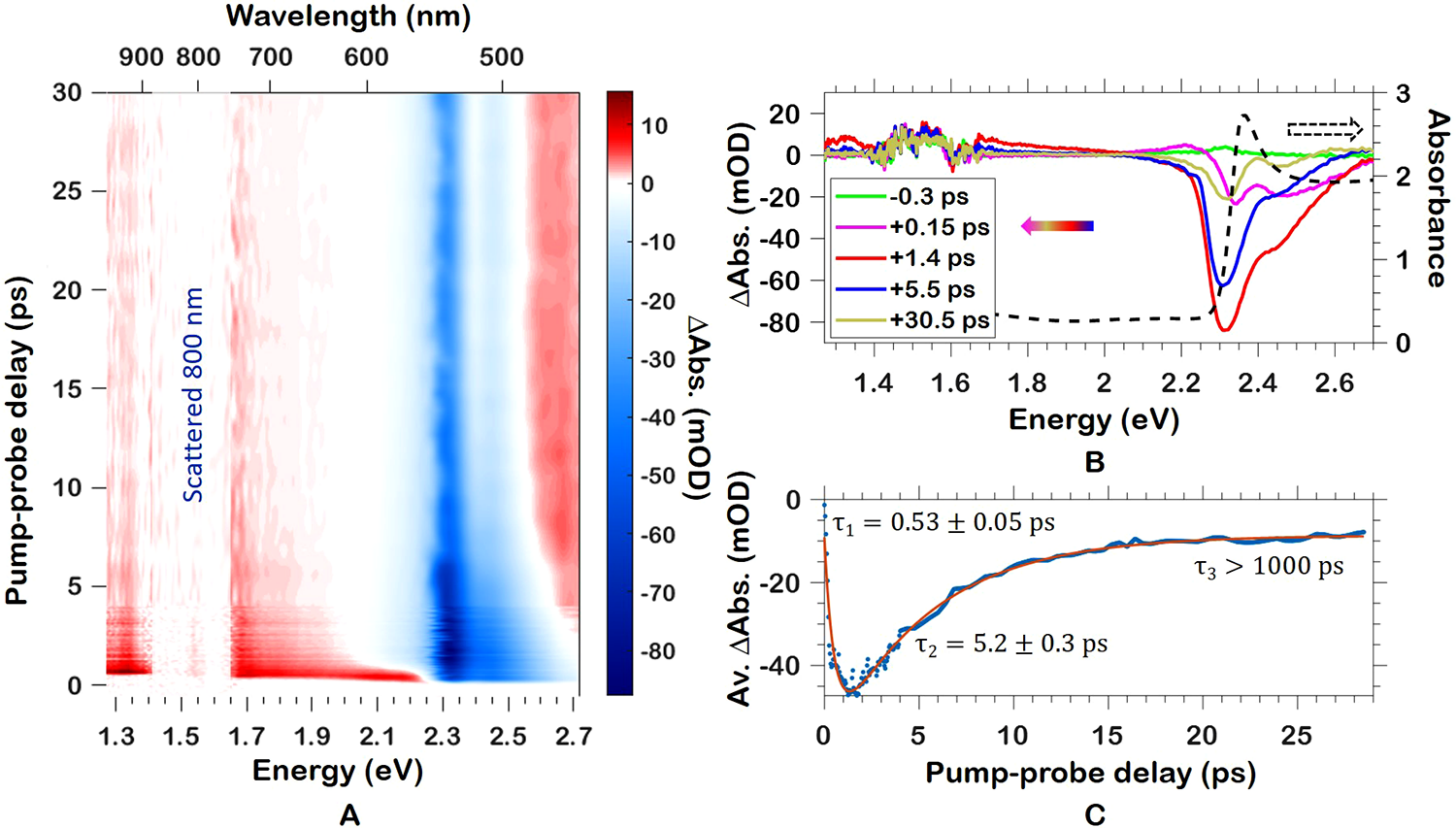
(A) Transient absorption spectrum, pumped at 400 nm to an initial carrier density of (2.1 ± 1.1)× 10^19^ cm^–3^. (B) Transient absorption line-out at several delay points. Scattered 800 nm (1.55 eV) light is responsible for the noise between 1.35 and 1.75 eV. (C) Spectrally averaged ground-state bleach at 520 nm (2.32 eV) (blue) as a function of pump–probe delay with a fit to a multiexponential function (red).

We now discuss how these processes relate to the transient SHHG, schematically illustrated in Figure 3, with results shown in Figure 4 and Figure S4. Figure 3A is a depiction of the visible pump–SHHG probe scheme. Figure 3B is a simplified schematic of the band structure and its interaction with the pump and probe pulse. As the first step, the pump pulse excites a fraction of the electrons from the valence band to the conduction band through single-photon excitation. These photons will cool and recombine radiatively or nonradiatively on ps-to-ns time scales. Second, the NIR probe electric field excites electrons from the valence band to the conduction band, where (third) during a single cycle of the laser-electric field, the electron and hole are accelerated, gaining energy until (fourth) the electron recombines with the hole, emitting a photon of much higher energy at an energy that is an odd-number multiple of the fundamental field energy.

**Figure 3:**
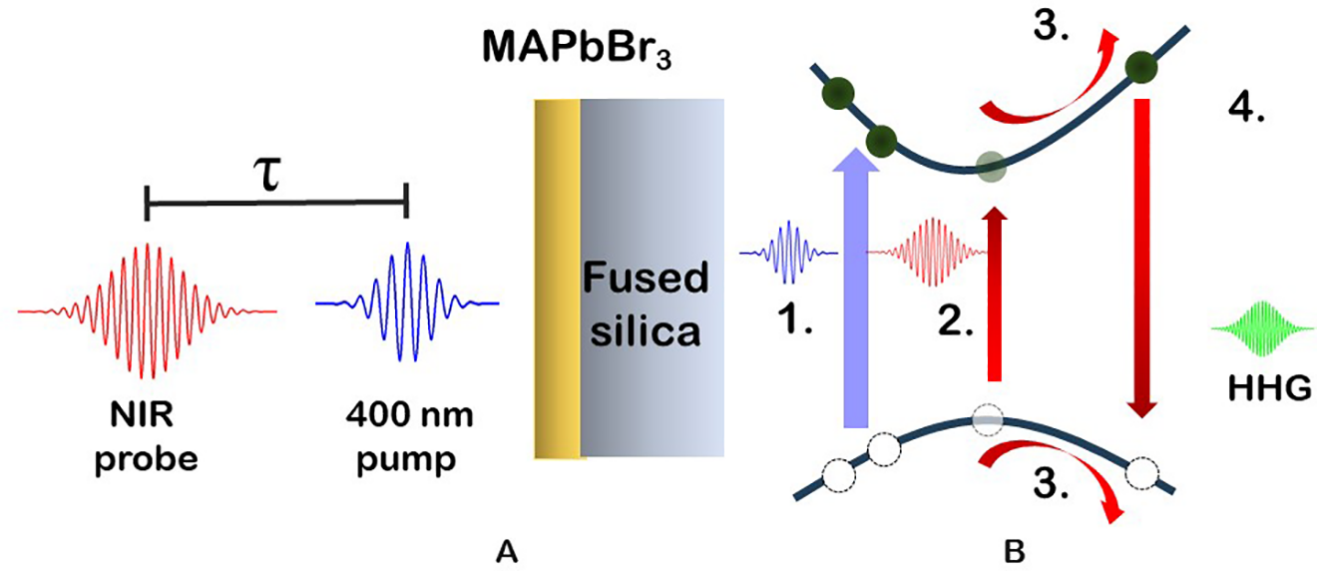
Depiction of the visible pump–SHHG probe scheme. (A) MAPbBr_3_ thin film on fused silica substrate (not to scale). The pump–probe delay τ is positive when the 400 nm pump arrives before the NIR driver. (B) Simplified schematic of the band structure and its interaction with the pump and probe pulse. (1) Pump pulse excitation. (2) Multiphoton excitation by a NIR pulse. (3) Electron and hole are accelerated. (4) The laser-driven current as well as the electron–hole recombination lead to HHG emission.

**Figure 4:**
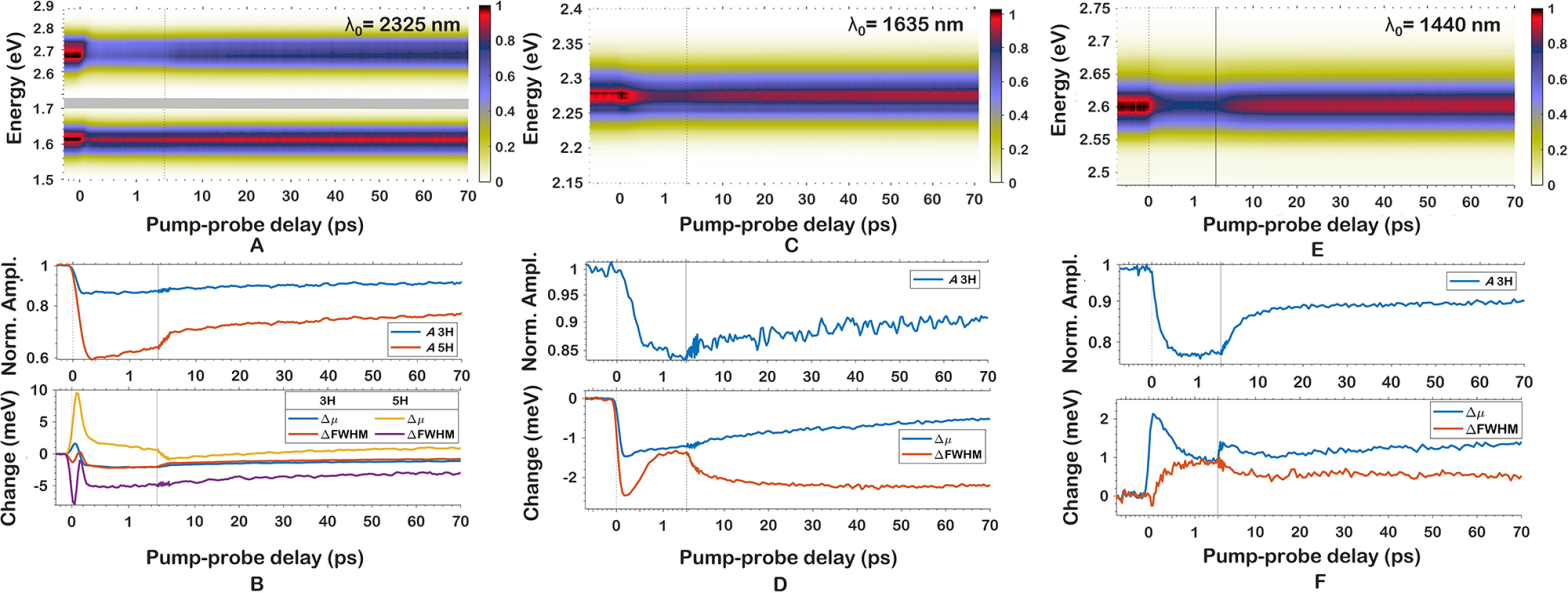
Pump–probe delays are depicted with different intervals before and after τ = 1.5 ps to enhance visibility of early pump–probe delay-dynamics. (A) Raw pump-on signal trace of the third (770 nm) and fifth (470 nm) harmonic of λ_0_ = 2320 nm. (B) Extracted amplitude and Δμ of panel A as a function of pump–probe delay. fwhm change is depicted in the same plot as Δμ. (C) Raw pump-on signal trace of the third (545 nm) harmonic of λ_0_ = 1635 nm. (D) Extracted amplitude, Δμ, and fwhm changes of panel C. (E) Raw pump-on signal trace of the third (480 nm) harmonic of λ_0_ = 1440 nm. (F) Extracted amplitude, Δμ, and fwhm changes of panel E.

To capture the varying transient SHHG signals and relate them to photophysical primary processes, a normalized Gaussian of the form *G*(ω) = 
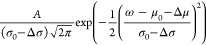
is fit to the frequencies ω of individual harmonic spectra shown in Figure 4. This enables tracking the transient evolution of the spectral width [full-width half-maximum Δfwhm = ], the amplitude *A*, and the transient change Δμ of the center frequency μ_0_ as a function of pump–probe delay. The resulting *A*(τ), Δμ(τ), and Δfwhm(τ) are then plotted and further analyzed. The fit of *A*(τ) has a 95%-confidence interval of ±0.002, while the errors in the fits of Δμ(τ) and Δfwhm(τ) increase from ±0.2 meV to ±0.8 meV with increasing photon energy due to the reduced number of bins per meV. The harmonic spectra for all combinations of λ_0_ and 400 nm pump were normalized, averaging *A* = 1, Δμ = 0 meV, and Δσ = 0 meV before pump–probe overlap. Under the experimental conditions of 0.3 mJ/cm^2^ pump fluence and 50 GW/cm^2^ probe peak intensity, the strong-field probe does not significantly affect the measured dynamics that were initiated by single-photon excitation process. This was verified by probing at different intensities. Transient changes can therefore be attributed to pump-induced processes affecting the high-harmonic generation alone.

The raw pump-on (*I*_on_(τ)) traces for the third and fifth harmonics of λ_0_ = 2320 nm, and the third harmonics of λ_0_ = 1635 and 1440 nm can be seen in Figure 4A,C,E, respectively. Each of the *I*_on_(τ) traces is normalized to the pump-off intensity at the same pump–probe delay (*I*_off_(τ)) to account for the wide range of central harmonic wavelengths and intensities as well as any fluctuations in the intensity. In Figure 4B,D,F, the *A*(τ), Δμ(τ), and Δfwhm(τ) as a function of pump–probe delay for λ_0_ = 2320, 1635, and 1440 nm, respectively, are depicted. Furthermore, transient SHHG spectra (*I*_on_(τ)/*I*_off_(τ) – 1) for all investigated λ_0_ can be seen in Figure S3. We fit *A*(τ), Δμ(τ), and Δfwhm(τ) again to *F*(τ)=∑_*i* = 0_^3^α_*i*_exp(−τ/τ_*i*_). To make sure that the fitting model works without issue, Δμ(τ) and Δfwhm(τ) are translated across the *y*-axis to obtain better fits as the value of these shifts does not matter for time scales. The fit results for *A*(τ), Δμ(τ), and Δfwhm(τ) are printed in Tables 1, 2, and S4, respectively These fits reveal that τ_*i*=1,2,3,GSB_, corresponding to the HCC, bi- and unimolecular recombination time scales extracted from the TA measurement, respectively, match well with their respective counterparts τ_1,2,3_ in the transient SHHG fitting. The values of τ_3_ from these fits were found to be >100 ps, similar to τ_3,GSB_, meaning that their values cannot be fitted with high accuracy due to the experiments focusing on earlier time and are therefore excluded from the tables.

**Table 1:** Extracted Transient-SHHG Fit Constants of *A*(τ) for Pump–Probe Traces in Figure 4^a^

λ_0_ and HO	τ_0_ and τ_1_ (ps)	τ_2_ (ps) Bi. rec.	*A* minimum
TA, GSB	IRF, 0.53 ± 0.05	5.2 ± 0.3	
2320 nm THG	IRF	2.2 ± 0.6	0.85
2320 nm 5HG	IRF	2.2 ± 0.2	0.6
1635 nm THG	0.41 ± 0.06^b^	1.4 ± 0.5	0.7
1440 nm THG	0.2 ± 0.1	4.9 ± 0.3	0.75

a The fitted values of τ_3_ are not enumerated as their values are unreliable.

b See text regarding the resonance of THG driven at λ_0_ = 1635 nm.

**Table 2:** Tabulated Δμ Shift Time Constants of Selected Harmonics^a^

λ_0_ and HO	τ_0_ and τ_1_ (ps)	τ_2_ (ps) Bi. rec.	Max.^b^ (meV)
2320 nm THG	IRF	3.9 ± 0.7	+2
2320 nm 5HG	IRF	3.9 ± 1.9	+10
1635 nm THG	IRF, 0.45 ± 0.9	0.48 ± 0.1	–1.5
1440 nm THG	0.8 ± 0.8, 1.1 ± 1.1	4.7 ± 0.7	+2, +1.4^c^

a Positive magnitude indicates a blueshift, negative magnitude a redshift. The fitted values of τ_3_ are not enumerated as their values are unreliable.

b Maximum value and magnitude of blue (positive)- or redshift (negative).

c Second apparent blueshift around +1.8 ps occurs due to narrowing of the spectrum.

The observed harmonic amplitude generally decreases immediately at temporal overlap. The initial decrease is obscured by the IRF with a fwhm of τ_0_ ≈ 0.095 ps, followed by a slower further drop to the amplitude minimum with time constant τ_1_ between 0.2 and 0.5 ps, corresponding to HCC to a Fermi–Dirac distribution. This two-component drop in harmonic amplitude appears differently for different wavelengths, but it can be seen clearly in Figure 4F. This shows that SHHG follows the transient photophysical primary processes—unlike in previously reported transient SHHG and -THG experiments, where the minimum amplitude is reached within or close to the IRF.^24,26,38^ The amplitude reaches a minimum value between τ = 0.5 and 1.5 ps but was also found to reach a minimum at longer values of τ higher pump intensities (Figure S6A). This reflects a slowing of the overall HCC rate, due to slower thermalization.^35,39^ Additionally, the suppression of the HHG yield is more pronounced for increased pump fluence (Figure S5A). This behavior was found to saturate at high intensities (Figure S5B). Furthermore, when increasing the pump fluence, the minimum of the amplitude occurs at later delays due to slower thermalization.^16,17,40^ We link this amplitude drop to a sudden reduction of available electrons in the valence band (VB) to participate in the SHHG process, resulting in depletion of the ground state in addition to excited-state blocking for above band gap harmonics. Simultaneously, a pre-excited carrier distribution has an effect on electron scattering and thus on electron–hole dephasing, as discussed in Figure 5. The carrier population in both conduction and valence bands is also initially out of thermal equilibrium, as will be elaborated on later.

**Figure 5:**
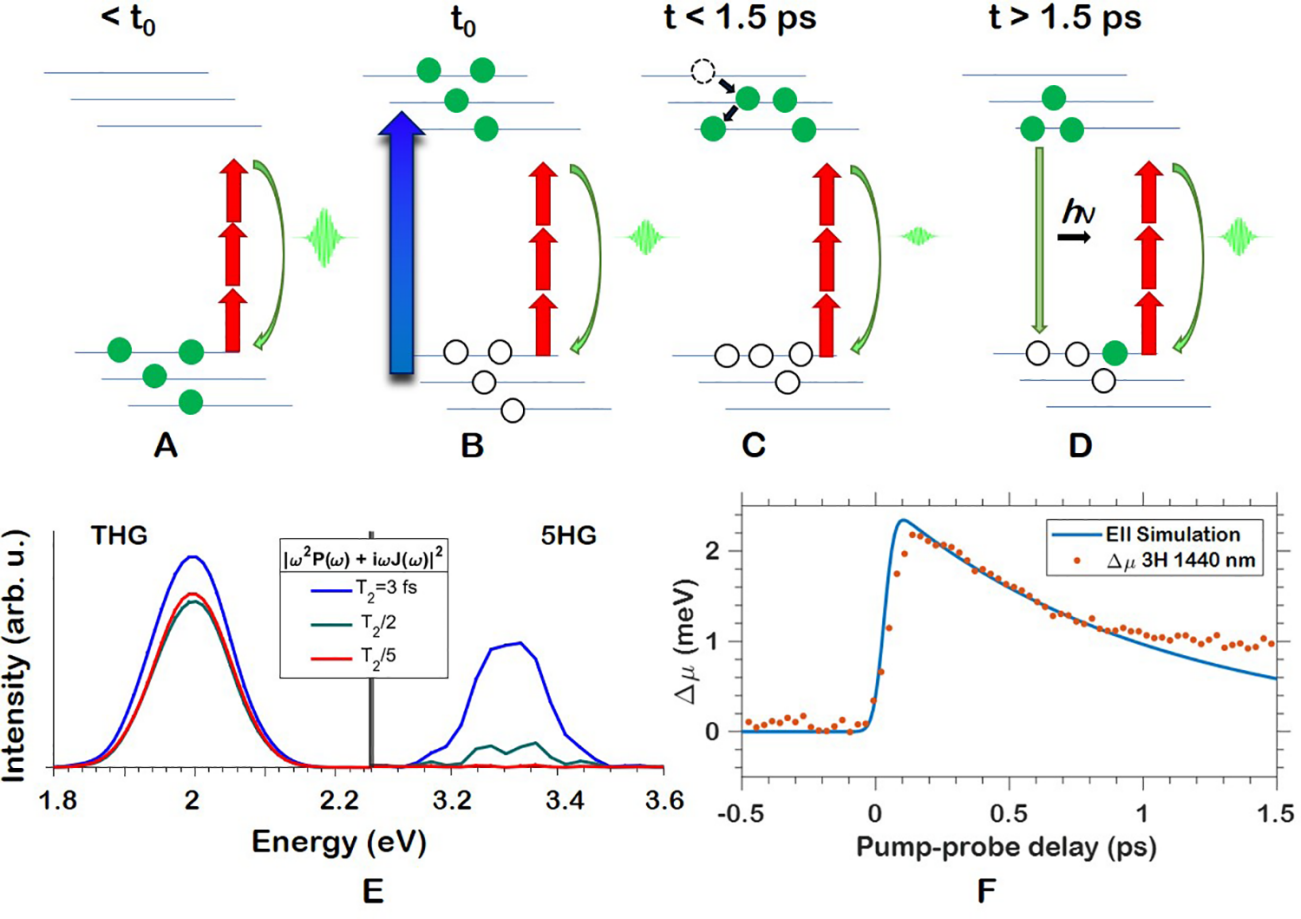
(A–D) Illustrations of process responsible for amplitude reduction and recovery in MAPbBr_3_. (A) THG process in MAPbBr_3_ in absence of carriers excited by a pump into CB. (B) At *t*_0_, excitation causes ground-state depletion and excited-state blocking, reducing the available carriers for HHG and thus decreasing the SHHG efficiency. (C) As thermalization of carriers to a Fermi–Dirac distribution occurs, minimal SHHG intensity is observed. (D) Bi- and unimolecular recombination occur and decrease the ground-state depletion and excited-state blocking effects by virtue of reducing the number of carriers. (E) SBE simulation of THG and 5HG in MAPbBr_3_ at λ_0_ = 1850 nm. Blue: *T*_2_ equal to half an optical cycle. Green, red: *idem*, with reduced *T*_2_ by a factor of 2 and 5, respectively. (F) Theoretical EII-induced blueshift from experimental parameters compared to the extracted center-frequency shift of the third harmonic of λ_0_ = 1440 nm.

The subsequent recovery of the harmonic intensity as a function of pump–probe delay can be linked to the aforementioned excited carrier recombination processes, namely, the bi- and unimolecular recombination,^37^ as more electrons become again available in the VB to participate in SHHG. Extracted time-scales for the amplitude recovery listed in the third and fourth column of Table 1 therefore match very well with the time-scales of the GSB recovery. Additional extracted amplitude drops and recovery constants can be seen in the Supporting Information in Table S2. The fitted values from the transient SHHG measurements for shorter IR driving wavelengths (Figure 4 and Table 1) match well with the TA measurements (Figure 2). For longer wavelengths, the fit values for τ_2_ are smaller, approximately 1/2 of those of shorter drivers. While this observation deserves further attention in a future study, we tentatively attribute this difference to the above vs below band gap emission of solid harmonic emission for different driving wavelengths. While above band gap emission, as it occurs for shorter-wavelength drivers, is more influenced by interband transitions, below band gap emission is rather linked to laser-driven intraband currents.^41^ As intraband currents as source of SHHG are more intimately linked to excited carrier populations, the bimolecular recombination effect may appear faster for below band gap HHG. Furthermore, it is likely that electron impact ionization (EII), as explained later on, interferes with the recovery at such short (<1.5 ps) time scales after temporal overlap.

A qualitative illustration of the process responsible for the amplitude drop and recovery is provided in Figure 5A–D. A standard THG process is depicted in Figure 5A as it would occur in the absence of a pump. In Figure 5B, as the pump and probe pulses overlap, the electron population is transferred from the VBs to the conduction bands (CBs) resulting in a nonequilibrium carrier distribution. However, as many of the electrons are still located in higher-lying CBs and have not thermalized through HCC, THG is not at its minimum as the lowest-lying CBs are not fully blocked from supporting the THG process. Figure 5C then depicts the HCC process reducing the number of immediately available carriers in the highest VBs, which matches closely to the minimum of the GSB in Figure 2. Figure 5D depicts one of the possible recombination processes that take place inside the perovskites, photoemission, as excited electrons recombine incoherently with holes in the VB, returning to pre-*t*_0_ equilibrium populations and increasing the number of emitters participating in the SHHG process. Early in the SHHG recovery, this is dominated by a bimolecular recombination process in which two electrons and one hole participate. Later on, it proceeds by more conventional electron–hole recombination which dominates when the electron population in the CB is insufficient for significant bimolecular recombination.

One of the possible mechanisms for the reduction in SHHG intensity around temporal overlap is that the excited carriers introduced in the CBs result in more rapid dephasing of the coherent carriers responsible for SHHG. Dephasing occurs due to scattering of the carriers with other carriers, phonons, and impurities and has an associated dephasing time *T*_2_. It is known that additional carriers excited from the VB through single-photon excitation increase scattering rates.^42^ To simulate this effect, the semiconductor Bloch equations (SBEs) for two conduction bands^43,44^ of MAPbBr_3_ along the R-Γ direction were solved for SHHG at 1850 nm.^45^ The SBEs yield an interband polarization *P*(ω) and an intraband current *J*(ω) for a given set of parameters, while the absolute square of their sum yields the intensity of the generated harmonic spectrum in the sample plane. The dephasing time in single-photon excitation (contrary to the strong-field driven HHG presented here) has been accessed experimentally,^42^ but for SBE simulations it was often assumed to be unusually fast. Recent works suggest this was necessary to adjust for imperfect electron–hole recollisions.^46^ Importantly, experimental photon-echo spectroscopy has shown that photoexcitation accelerates dephasing.^42^ In our simulation, dephasing was set to half the optical cycle duration (∼2.5 fs), depicted in the blue curve of Figure 5E, and is then reduced by a factor of 2 (∼1.25 fs, green curve) and five (∼0.5 fs, red curve), following the above stated observations from photon-echo spectroscopy. The SBEs clearly show that reducing the *T*_2_ has a deleterious effect on the amplitude of the third and fifth harmonic, but more so on the fifth harmonic. Within the analytical two-band description of SHHG,^27^ dephasing acts as an window function for reducing the amplitude of the interband polarization and intraband current, which in turn means that a shorter dephasing imprints an amplitude Fourier filter onto the HHG spectrum that reduces the overall emission strength. In the experimental traces in Figure 4A,C,E, one can see an instantaneous drop, which is linked to the T_2_ reduction and GSB through the addition of incoherent carriers as well as a slower component which corresponds to HCC. Our simulations and experiments thus show that an increase in incoherent carrier population by photoexcitation reduces the amount of available carriers for HHG. This affects the emissions strength and is accompanied by a reduced electron–hole coherence, which has the same effect of an amplitude reduction on the HHG emission strength. Both effects are likely at play for the early time-scale emission suppression observed in the present work.

Time-constants and magnitudes for either Δμ shifts or amplitude changes do not seem to depend on the μ_0_ of a given harmonic. The fifth harmonic of 2320 nm (465 nm) and the third harmonic of 1440 nm (480 nm) have similar photon energies, and the former only experiences a slightly higher static absorbance (Figure 1A), while TA effects (Figure 2A) are also comparable at these wavelengths. It is therefore interesting to note that the 2320 nm 5HG experiences a much faster amplitude drop and blueshift compared to 1440 nm THG. We attribute this to the much stronger effect of reduced dephasing on higher harmonic generation processes as demonstrated by the SBE simulation.

The Δμ shifts of the center frequency occur on similar time-scales as the amplitude changes because both effects are driven by incoherent carrier populations. Several effects interplay and affect the observed center frequency shift. The most important cause for blueshifts after temporal overlap is electron impact ionization (EII) seeded by the already excited CB-population. In EEI for our experiment, the 400 nm pump pulses promote electrons into the conduction bands. The pump-induced electron–hole plasma acts as a seed population for EII driven by the IR probe laser pulse, which can now trigger EEI by single-photon absorption of the electron–hole plasma. During EII, the large number of electrons excited from the VB cause a negative change to the index of refraction. This negative change in refractive index in turn causes a phase accumulation of the NIR fundamental, which translates to its harmonics. Larger changes are expected for longer wavelengths and higher harmonics as the EII blueshift is proportional to the wavelength and multiplies with harmonic order.^47^

EII is responsible for most blueshifts of Δμ, as summarized in Table S3. Using the pulse durations measured through frequency-resolved optical gating of pump (∼0.055 ps) and probe (∼0.065–0.07 ps), respectively, we find a good match for the blueshift of 1440 nm data in Figure 4F as can be seen in Figure 5F, showing a qualitative agreement between the modeled and measured blueshift time-scales. Similar to the amplitude drop, the Δμ shift becomes larger as a function of pump fluence, as can be seen in Figure S6B. Overall, Figure S6 confirms that two linked but separate mechanisms are responsible for the observed Δμ(τ) and *A*(τ) trends as the center frequency shift is insensitive to the excited-state blocking and is only sensitive to the total carrier population. The Δμ recovery after the initial shift is still linked to the recombination dynamics through the extracted time-scales. Due to the high carrier density created by the pump, the transient real and imaginary refractive index change and their effect on Δμ cannot be neglected around the bandgap,^48^ which is laid out in more detail in the Supporting Information and Figure S2A,B. This effect is minor compared to EII, but it becomes large around the bandgap, where it is possibly responsible for the observed redshift instead of blueshift in the base of the third harmonic of 1635 nm. One notable change is a negative-to-positive refractive index change around 2.4 eV, which is responsible for blue to redshift of Δμ on the same time scale for the third harmonic of λ_0_ = 1560 nm depicted in Figure S4B. The fwhm of the various SHHG spectra changes on comparable time scales to Δμ(τ) and *A*(τ). As the center frequency is shifted through EII, with matching maxima of both Δμ(τ) and Δfwhm(τ), while the amplitude changes through HCC and remains somewhat flat as the states at the bottom of the CB and top of the VB reach a thermal equilibrium, the fwhm changes according to the interplay of both and is therefore not directly linked to a single physical process.

We note that the HHG amplitude changes are generally much stronger than the TA signals (Figure 2), which suggests up to 20% increase of transmission right at the band gap at 530 nm (2.3 eV) and a maximum decrease of up to 5% between 600 and 700 nm (1.65–2.25 eV). This suggests that the HHG amplitude is not strongly affected by the changes of the complex-valued refractive index. Also changes in the real part should not affect the HHG amplitude, due to the low sample thickness, below the wavelength of the emitted light. However, the refractive index changes are captured in the center frequency shifts and broadening of the harmonics. As such, measuring center frequency shifts and amplitude changes in transient SHHG simultaneously provides an elegant way of tracing both state blocking and dephasing changes through amplitude and refractive index changes through center frequencies. The sensitivity to state blocking via frequency shifts of harmonics may allow measurement of wavelength-resolved primary processes such as recombination rates, similar to transient absorption.

A further specific observation worth addressing is that, for λ_0_ = 1635 nm in Figure 4C, the third harmonic amplitude maximum *increases* by 5–10% for the first 0.2 ps after temporal overlap, combined with a narrowing of the fwhm and a minor Δμ shift. This results in an apparent enhancement, although the total number of counts still decreases as a function of pump–probe delay, as depicted in Figure 4D. The third harmonic of λ_0_ = 1635 nm (545 nm) is resonant with the onset of the exciton absorption feature (Figure 1A). The positive feature forms rapidly upon excitation and matches the estimated lifetime of the exciton,^49^ although exciton dissociation is likely due to the excess carriers in the CBs. The transient spectrum of the THG of 1635 nm in Figure S3D in the Supporting Information shows the feature clearly as well. Thus, time-resolved HHG spectroscopy can provide a new way to study the transient formation and dissociation of excited states such as excitons. In this particular case, it is likely that the exciton enhances both multiphoton excitation, as the exciton is three-photon resonant, as well as the radiative interband polarization, often thought of as electron–hole recollision.

In conclusion, we conducted static and transient SHHG measurements at NIR driving wavelengths in MAPbBr_3_ and established a clear link to the ultrafast carrier dynamics and time scales at high carrier densities, verified independently by optical transient-absorption spectroscopy. We have shown that these high carrier densities result in significant transient changes in both amplitude and center frequency of the harmonics and explained these changes through EII and ground-state depletion and excited-state blocking as well as dephasing. We have demonstrated how these processes influence HHG, which allows linking the HHG signals to the ultrafast dynamics of MAPbBr_3_, namely, HCC as well as bi- and unimolecular recombination. The narrowing of the third harmonic at resonance is explained by the rapid formation and dissociation of the exciton at this energy. The transient SHHG signal reduction, as observed in our study, can be rationalized by the increase in carrier density. Near-complete (Figure S5) femtosecond signal extinction adds a new method of optical control: This capability has significant implications for future research and applications in ultrafast optics, as it opens the door to manipulating and controlling harmonic emission with unprecedented precision and speed. This has implications not only in fundamental research but also in practical applications such as ultrafast optoelectronic devices. This further implies that all-optical control of SHHG may be possible in other APbX_3_ perovskites and can likely be extended to other semiconductor materials and their photophysical primary response of the valence electrons. Potential improvements to optical control could be made by tuning the pump-wavelength to the bandgap as hot-carrier cooling occurs on faster time-scales when pumped closer to resonance.^40,50^

## Supporting Information Available

Sample preparation and characterization, experimental methods for both SHHG and transient absorption measurements, fitting routines, additional transient and static spectra, figures showing saturation and intensity effects as well as tables with additional extracted parameters (PDF)
Transparent Peer Review report available (PDF)
