# Supplementary material for: Transient High-Harmonic Spectroscopy in an Inorganic–Organic Lead Halide Perovskite
Source: J Phys Chem Lett. 2023 Nov 28;14(48):10810–8. doi: 10.1021/acs.jpclett.3c02588 (PMC10711791; doi:10.1021/acs.jpclett.3c02588)
Supplement: Supplementary file 1 — jz3c02588_si_001.pdf [file jz3c02588_si_001.pdf]

Supporting Information: Transient High-harmonic Spectroscopy in an Inorganic-organic Lead Halide Perovskite

Maarten L.S. van der Geest,[†] Jeroen J. de Boer,[‡] Kevin Murzyn,[†] Peter Jürgens,[†,¶] Bruno Ehrler,[‡] and Peter M. Kraus[*,†,§]

†*Advanced Research Center for Nanolithography (ARCNL), Science Park 106, 1098 XG, Amsterdam, The Netherlands.*

‡*Center for Nanophotonics, AMOLF, Science Park 102, 1098 XG, Amsterdam, The Netherlands.*

¶*Max-Born-Institute for Nonlinear Optics and Short Pulse Spectroscopy, D-12 489 Berlin, Germany*

§*Department of Physics and Astronomy, and LaserLaB, Vrije Universiteit, De Boelelaan 1105, 1081 HV Amsterdam, The Netherlands.*

E-mail: P.Kraus@arcnl.nl

Sample Preparation and Characterization

Sample preparation was based on an earlier reported preparation with minor adjustments.[1] A brief summary with all necessary steps is provided here.

Quartz substrates (12×12 mm^2, with 1 mm thickness, as well as 16×14 mm^2, with 1 mm thickness) were cleaned with water and soap, followed by ultrasonication in deionized water, acetone and isopropyl alcohol. Finally, an O_2-plasma cleaner was used on the

substrates for 15 min. A spincoating procedure was then used to produce the $MAPbBr_3$ thin film. The precursor solution consisted of 1.1 mol/L methylammonium bromide (98.0%, Sigma-Aldrich) and 1.1 mol/L lead(II) bromide ($PbBr_2$ $>$ 98.0%, TCI), dissolved into a 4:1 dimethylformamide (anhydrous, 99.8%, Sigma-Aldrich)/dimethyl sulfoxide (anhydrous, $\geq$ 99.9%, Sigma-Aldrich) solvent mixture. A hot plate was used for overnight dissolution at 60 °C The $MAPbBr_3$ precursor solution (150 µL) was spun onto the quartz substrates at 6000 rpm for 30 s after cooling. Fifteen seconds after the start of the spincoating, 150 µL of chlorobenzene antisolvent (anhydrous, Sigma-Aldrich) was added to the substrate. The substrates were annealed at 100 °C for 1 h after the spincoating. The entire spincoating procedure was carried out inside a glovebox maintained at an O_2 level below 1 ppm.

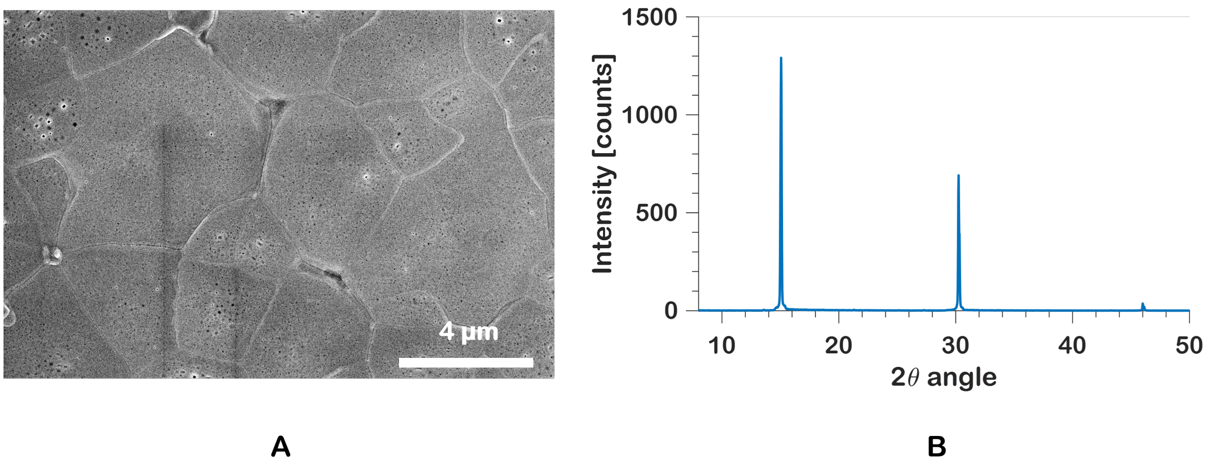

Figure S1: A) Scanning electron microscope micrograph of a representative $MAPbBr_3$ sample area. B) XRD spectrum showing characteristic peaks confirming the sample is crystalline cubic $MAPbBr_3$.

The sample was characterized using XRD to confirm crystallinity and scanning electron microscopy was used to estimate the crystal domain size and judge sample quality. The average crystal domain size based on several XRD micrographs is estimated to be between 1 and 8 µm. X-ray diffraction (XRD) measurements were conducted using a D2 Phaser (Bruker) with a copper K-α_2 (8027.8 eV) source. Ultraviolet-visible (UV-Vis) absorption spectroscopy measurements were conducted with a UV-Vis spectrophotometer (*UV*-2600,

Shimadzu) with a 1-nm step size, specially adapted to measure solid samples.

Experimental Details

Solid high-harmonic generation

The setup used in the presented experiments is slightly adapted from a previously reported transient absorption setup, using the same laser and stages.[2] Briefly, an 800-nm, 45-fs, 3.5-mJ output is generated at a repetition rate of 2 kHz using a Solstice ACE (Spectra Physics) laser system. The output is split at the laser exit, where part of the beam is redirected into an optical parametric amplifier (OPA) and the other part is used for generating 400-nm pump pulses. The 400-nm pulses are generated through second harmonic generation of 800 nm in a β-barium borate (BBO, $\theta = 29.8°$) crystal. The 800-nm fundamental is filtered from the pump using a zero-degree high reflector and 400 nm bandpass filter. The 400-nm pump is then attenuated through a neutral density filter wheel. The pump is spatially overlapped with the probe using a pinhole that can be moved into the sample plane. The near infrared (NIR) probe is generated inside an OPA system (*TOPAS*, Light Conversion) and subsequently delayed using a delay stage (M-531.DD1, PI), after which it is sent unto the sample. The NIR and any generated harmonics are then sent into an optical fiber connected to a spectrometer (*Maya* 2000 *Pro*, Ocean Optics). The noncollinear angle of 6° allows for spatial filtering of the direct pump beam, although scattered light can still reach the spectrometer and obscures harmonics in the range between 390 and 420 nm a number of measurements. Temporal overlap is found using difference frequency generation (DFG) of the NIR and 400-nm fields in a second BBO crystal ($\theta = 29.8°$), mounted in the same plane as the sample, yielding difference frequencies of between 480 nm (400 −2300 nm) to 580 nm (400 −1300 nm) at temporal overlap. The infrared driver was attenuated through the use of a second neutral density filter wheel. The cross-correlation of the NIR and 400-nm fields gives the temporal resolution of each combination of wavelengths, which was found

to be between 95 and 110 fs at full-width at half-maximum (FWHM). Parallel polarization between the 400-nm pump laser and NIR probe was ensured by inserting a broadband NIR $\lambda/2$-waveplate in the probe beampath.

The carrier density excited by the pump and its excited-state fraction were estimated by assuming a 1:1 photon to excitation conversion in addition to a $> 99\%$ absorption of the pump-pulse. The fluence was determined to be $0.3\,\mathrm{mJ/cm^2}$ by measuring the power at the entrance of the setup, the transmission through the setup and determining the spotsize with a knife-edge measurement. The total number of states is determined by the total number of unitcells within one $\mathrm{cm^3}$, which was found to be $4.5 \times 10^{21}\,\mathrm{cm^{-3}}$ based on a $3.582\,\mathrm{g/cm^3}$ density.[3]

Table S1: Table of FWHM spotsizes for intensity measurements in Fig.1B,C,D.

Wavelength	FWHM Spotsize	Error
1260 nm	$1.7 \times 10^{-4}\,\mathrm{cm^2}$	$\pm 0.5 \times 10^{-4}\,\mathrm{cm^2}$
1440 nm	$2.1 \times 10^{-4}\,\mathrm{cm^2}$	$\pm 0.5 \times 10^{-4}\,\mathrm{cm^2}$
1570 nm	$1.8 \times 10^{-4}\,\mathrm{cm^2}$	$\pm 0.5 \times 10^{-4}\,\mathrm{cm^2}$
1630 nm	$2.1 \times 10^{-4}\,\mathrm{cm^2}$	$\pm 0.5 \times 10^{-4}\,\mathrm{cm^2}$
1850 nm	$1.7 \times 10^{-4}\,\mathrm{cm^2}$	$\pm 0.5 \times 10^{-4}\,\mathrm{cm^2}$
2100 nm	$1.9 \times 10^{-4}\,\mathrm{cm^2}$	$\pm 0.5 \times 10^{-4}\,\mathrm{cm^2}$

Intensity scalings for the various infrared driver wavelengths were conducted in the same setup by simply blocking the pump and attenuating the infrared driver using a ND filter. The laser power was measured using a powermeter ($S401C$, Thorlabs), while spotsizes were measured at the sample position using a beamprofiler (*Beamage*-$4M$, gentec). Measured spotsizes are provided in table S1.

The obtained vacuum intensity I_{vac} was then converted to the intensity inside the material through taking into account the strong Fresnel reflection and large refractive index.[4,5] This results in Equ. S1 relating the intensity inside the material to the vacuum intensity.[4]

$$I_{mat} = n(\lambda_0)\left(\frac{2}{1+n(\lambda_0)}\right)^2 I_{vac} \tag{S1}$$

As a dielectric, the quartz substrate also has the capability of generating harmonics, but due to its unfavorable thickness affecting phase matching in addition to its much larger bandgap, the counts from the substrate are lower by a factor of more than one-hundred for all harmonics and the measured harmonic signal and changes thereof are therefore attributed to the perovskite thin film. Furthermore, the excitation pump is not intense enough to excite significant carrier population from the valence band to the conduction band of quartz through multiphoton excitation.

Transient SHHG spectral and temporal fitting

As the various harmonics investigated range in wavelength from 770 to < 300 nm, a conversion of the harmonic spectrum from wavelength to eV was done to make counts more comparable.[6] Harmonics of λ_0 are separated from each other due to their orders of magnitude difference in individual counts and subsequently scaled to their respective average intensities before $\tau = 0\,\mathrm{ps}$ (taken as having an average amplitude of 1). Each individual harmonic is then fit to a normalized Gaussian, given by Equ.S1, so that the amplitude, FWHM and center frequency changes can be tracked and extracted as a function of pump-probe delay for every delay step.

$$G(\omega) = \frac{A}{(\sigma_0 - \Delta\sigma)\sqrt{2\pi}} \exp\left(-\frac{1}{2}\left(\frac{\omega - \mu_0 - \Delta\mu}{\sigma_0 - \Delta\sigma}\right)^2\right) \tag{S1}$$

Where $2\sqrt{2\ln(2)} * \sigma_0$ is the FWHM, and μ_0 is the center frequency without pump pulse, while μ tracks the center frequency change. The amplitude A is a scaling factor to measure the change in amplitude as a function of pump-probe delay. As long as the integral of the spectrum can be well approximated with an error function, the center frequency and amplitude can be tracked with a high degree of accuracy using Equ. S1. At very high carrier densities ($>1 \times 10^{20}\,\mathrm{cm}^{-3}$), where photobleaching and dielectric breakdown can occur, this model might fail to fit the spectrum as the $\Delta\mu$ shift originating from the electron impact

ionization (EII) can cause the harmonic spectrum to no longer resemble a Gaussian for every λ_0 near infrared driver.

Exciting $MAPbBr_3$ above its bandgap causes significant photoluminescence (PL) to be observable by eye. Photo-emission through visible PL is one of the recombination mechanisms in which electrons in the conduction band recombine with holes in the valence band and become available again for SHHG. The PL signal can be faintly observed on the spectrometer during pump-probe delay measurements, but as PL follows a wide-angle distribution,[7] while the harmonics are collinear with the NIR driving field, the measured intensity of the PL was insignificant compared to the intensity of third harmonics in the same spectral region, such as the third harmonic of 1560 nm. It's amplitude is therefore not taken into account.

Transient Absorption

Time-resolved visible transient absorption spectroscopy was conducted on a separate setup, adjusted from earlier reports.[8] A titanium:sapphire laser (Spectra-Physics Solstice Ace, 800 nm, 2 mJ/pulse, 100 fs FWHM, at 3 kHz repetition rate) was used as source. Fifty percent of the output is sent towards a BBO crystal for frequency doubling to 400 nm. The 400-nm pump pulses are attenuated through detuning the BBO. Five percent of the 800-nm beam (100 µJ) is passed through an computer-controlled adjustable delay stage, attenuated using a neutral density filter, and focused a small distance behind a 1-mm sapphire plate to generate the white light probe beam via supercontinuum generation. A chopper is synchronized to the beam to block every other pulse for the transient absorption experiment. The spectrum of the white light is measured when the pump is transmitted (I_p) and the pump is blocked (I_0) using a single diode array (Hamamatsu NMOS S3901-512Q). Fast electronics (TEC5) were used for reading out the signal. The difference absorption ΔA is calculated using $\Delta A = -log_{10}(I_p/I_0)$. A half-wave plate was used to match the relative polarization between the pump and the probe. The instrument response is 200 fs. A Fourier filter was used to remove rapidly oscillating components originating from electronic noise in the spec-

tral domain. The pump intensity is estimated using a pinhole measurement by measuring the power through a pinhole as a function of position along the beam propagation direction.

The Burstein-Moss shift of the GSB in a transient absorption spectrum can be used to estimate carrier densities at high excitation levels and is described by Equ. S1.[9,10]

$$\Delta E_{BM} = \frac{\hbar^2}{2m_r^*}(3\pi^2 n_0)^{2/3} \tag{S1}$$

The shift ΔE_{BM} is taken as the difference between the FWHM of the TA at the minimum and at +30 ps. It follows from Equ. S1 that a measured FWHM difference ΔE_{BM} of 150 meV and a reduced mass of the electron hole-pair of $m_r^* = 0.185 m_e$,[11,12] that the carrier density is $(2.1 \pm 1.1) \times 10^{19}\,\mathrm{cm}^{-3}$. The uncertainty mainly originates in the range of reported effective hole-pair masses between 0.09 and 0.25 m_e.[11–15]

Transient complex refractive index

One possible factor in changes in the transient SHHG is a change in the transient Fresnel reflection as a consequence of changing n and k. Using a Kramers-Kronig (KK) transformation, through a method published by Ashoka *et al.*,[16] and the previously reported n and k as additional constraints,[17] the transient dielectric constants $\Delta\epsilon_1$ and $\Delta\epsilon_2$ can be obtained. Through a conversion, Δn and Δk can then be obtained from $\Delta\epsilon_1$ and $\Delta\epsilon_2$. The results are depicted in Fig. S2.

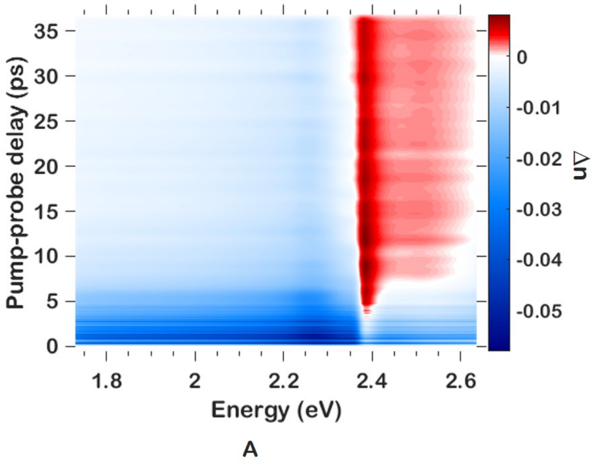

Figure S2: A) Δn as a function of pump-probe delay, extracted from the transient absorption and static absorption spectra through a KK transformation. B) Δk as function of pump-probe delay.

These results show that the transient reflection changes originating from pump-induced changes to n and k are not zero, but also do not change dramatically and are not the main cause of amplitude changes. They do however affect the apparent blue- and red-shift around the bandgap where gradients of n and k affect both absorption and reflection.

Additional transient solid HHG data

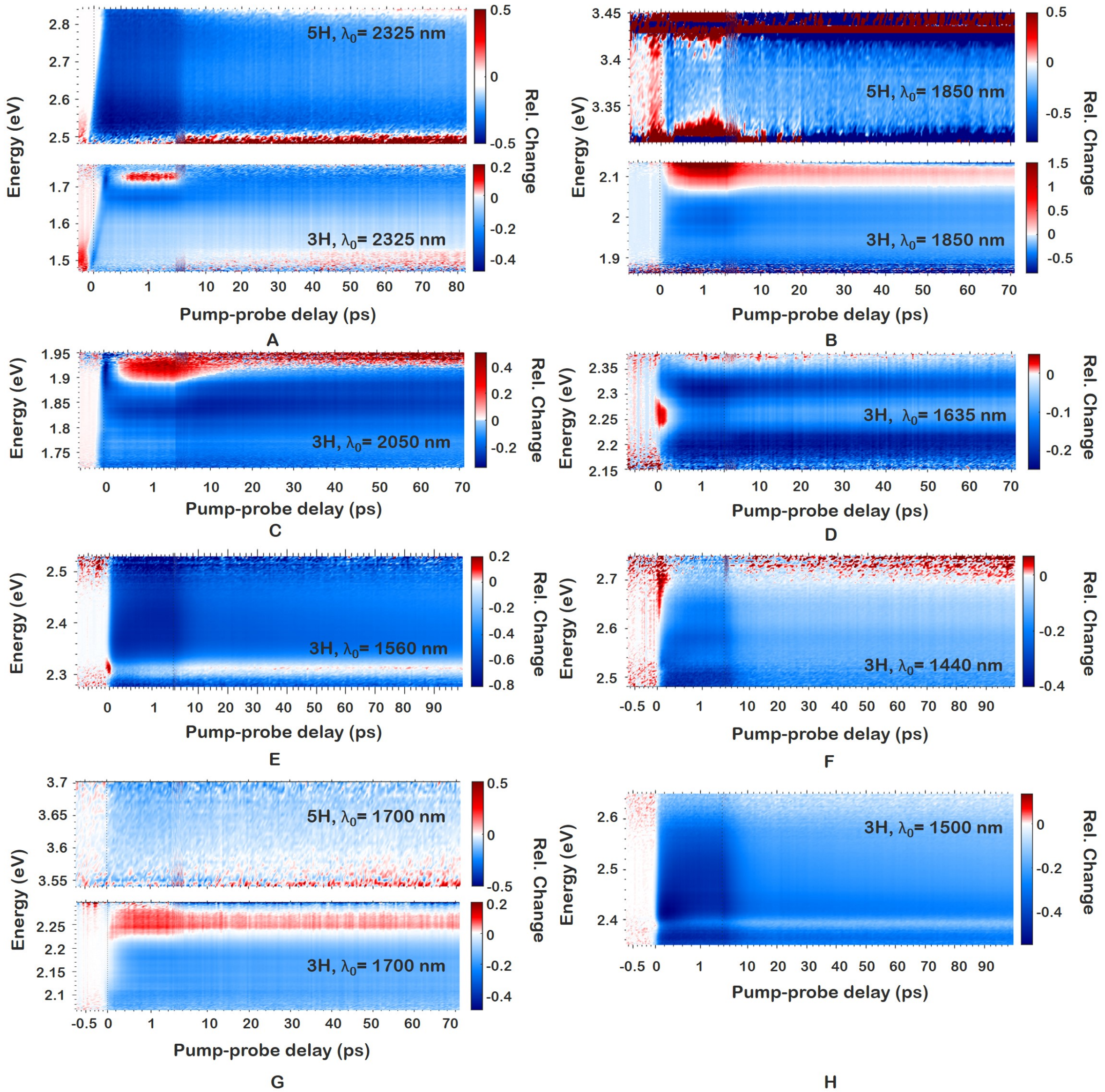

Figure S3: Relative (Rel.) changes of spectral components for harmonics of various NIR λ_0. A change of 0 equals no change between pump on and pump off, while positive and negative changes are depicted A) Transient of the third (770 nm) and fifth (470 nm) harmonic of $\lambda_0 = 2320$ nm. B) Transient of the third (618 nm) and fifth (310 nm) harmonic of $\lambda_0 = 1850$ nm. C) Transient of the third (683 nm) harmonic of $\lambda_0 = 2050$ nm. D) Transient of the third (545 nm) harmonic of $\lambda_0 = 1635$ nm. E) Transient of the third (520 nm) harmonic of $\lambda_0 = 1560$ nm. F) Transient of the third (480 nm) harmonic of $\lambda_0 =1440$ nm. G) Transient of the third (567 nm) and fifth harmonic (340 nm)of $\lambda_0 = 1700$ nm. H) Transient of the third (500 nm) harmonic of $\lambda_0 = 1500$ nm.

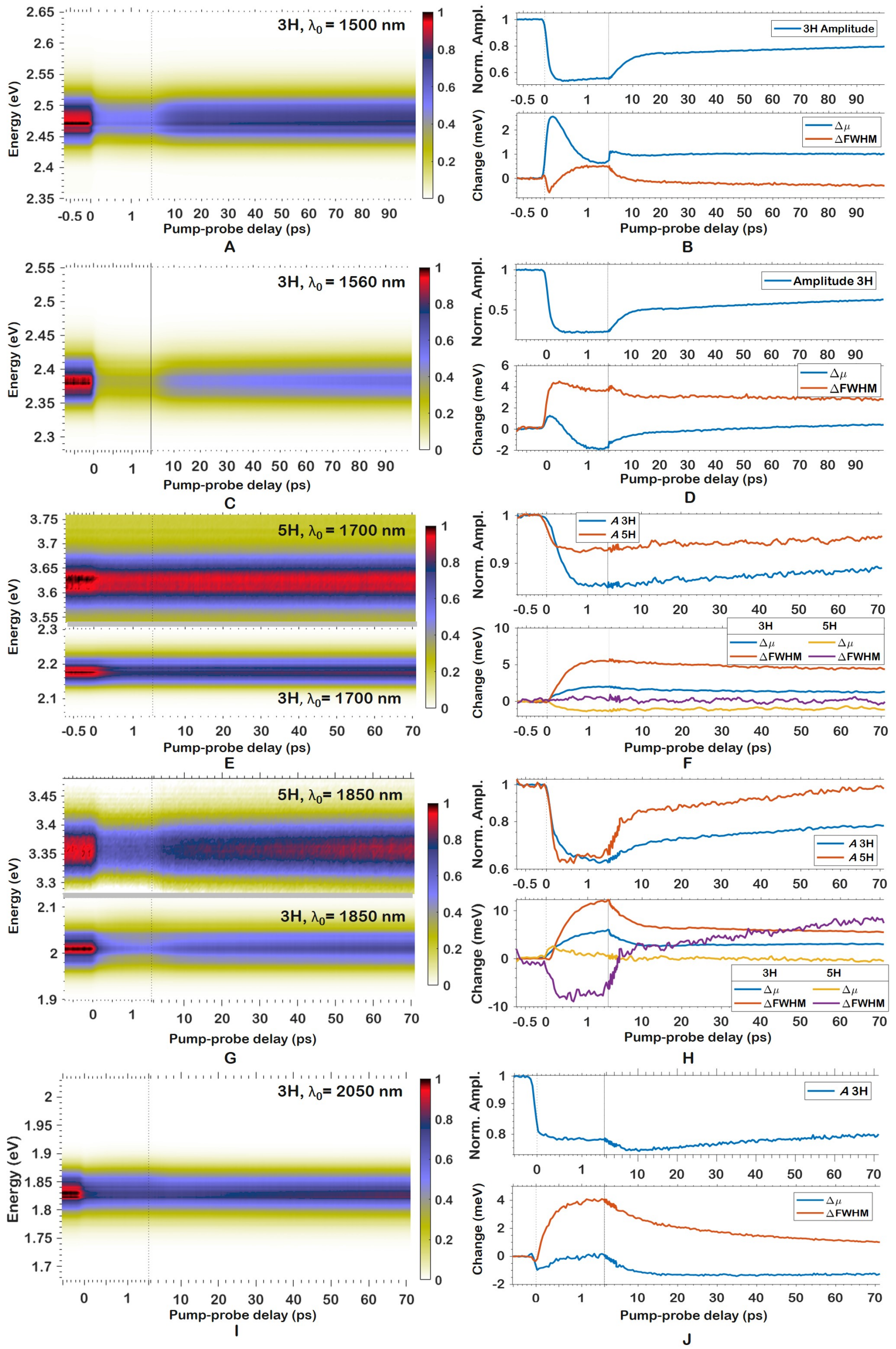

Figure S4: Accompanying figures to Fig. 3 in the main text. A) Transient SHHG of the third (500 nm) harmonic of $\lambda_0 = 1500$ nm. B) Extracted Amplitude, $\Delta\mu$ and ΔFWHM for A). C) Transient SHHG of the third (520 nm) harmonic of $\lambda_0 = 1560$ nm. D) Extracted Amplitude, $\Delta\mu$ and ΔFWHM for A). E) Transient SHHG of the third (580 nm) and fifth (340 nm) harmonic of $\lambda_0 = 1700$ nm. F) Extracted Amplitude, $\Delta\mu$ and ΔFWHM for C). G) Transient SHHG of the third (618 nm) and fifth (370 nm) harmonic of $\lambda_0 = 1850$ nm. H) Extracted Amplitude, $\Delta\mu$ and ΔFWHM for E). I) Transient SHHG of the third (683 nm) harmonic of $\lambda_0 = 2050$ nm. J) Extracted Amplitude, $\Delta\mu$ and ΔFWHM for G).

Amplitude and center frequency changes

This section depicts figures to show intensity dependence for two third harmonics of two infrared wavelengths. Fig. S5 shows the effect that pump intensity has on the amplitude of the third harmonic of 1560 nm at a fixed delay of +0.4 ps. In general, a suppression or even a slight recovery for a given delay is observed as a function of intensity. Fig. S6 shows the amplitude and center frequency dependence of the third harmonic of 1850 nm at four different intensities. This figure shows that the minimum shifts as a function of intensity. We attribute this to HCC rates slowing down as a function of pump intensity. The figure also shows that the center frequency shift reaches a maximum at a different point than the amplitude, which is evidence for two different mechanisms working concurrently.

The transient saturation in Fig. S5 is fully reversible. Absence of photodegradation was verified by moni-toring and verifying the stability of luminescence from the samples.

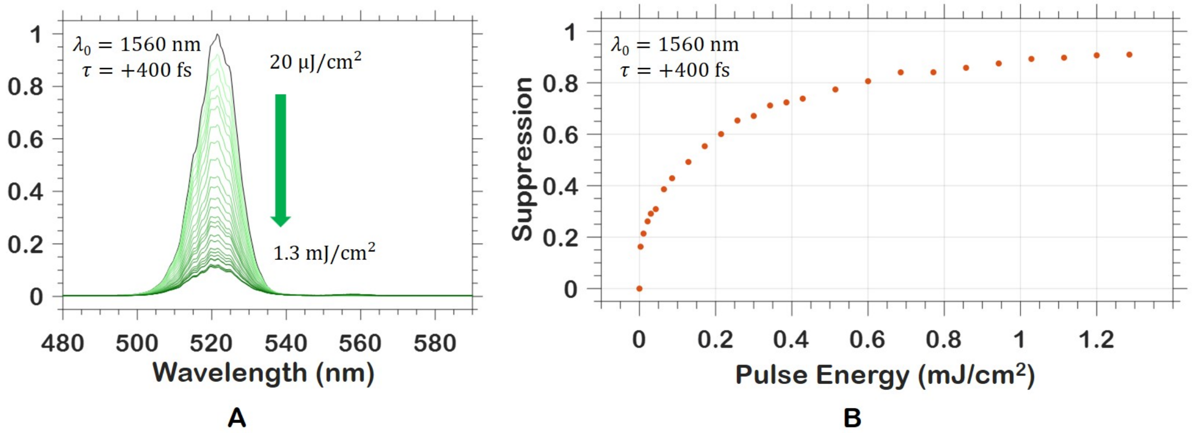

Figure S5: A) Spectral amplitude as function of pump intensity for the third harmonic of $\lambda_0 = 1560$ nm at $\tau = 0.4$ ps fs. B) Suppression $(1 - A/A_0)$ as a function of pump intensity

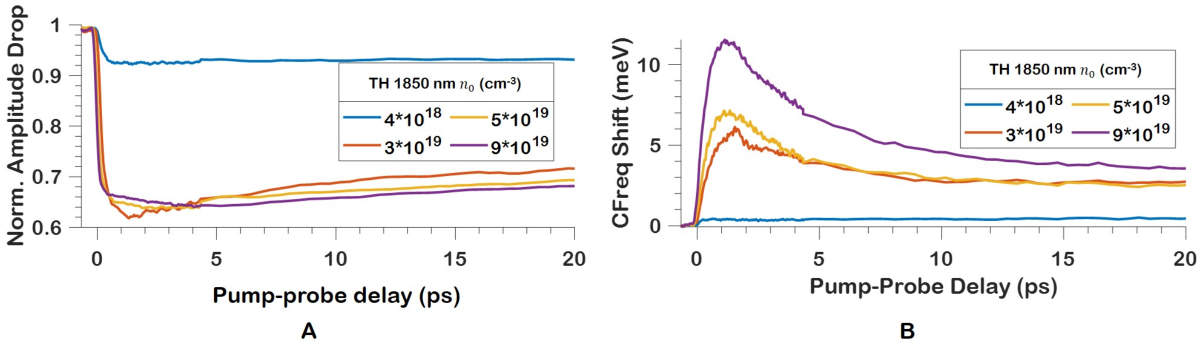

Figure S6: A) Amplitude changes at four intensities for the third harmonic of 1850 nm as a function of pump-probe delay. B) Center frequency changes at four intensities for the third harmonic of 1850 nm as a function of pump-probe delay.

Additional Tables

This section depicts the complete tables of extracted amplitude (Table.S2), center frequency (Table.S3) and FWHM (Table.S4) changes for eight NIR wavelengths and their various visible harmonics.

Table S2: Amplitude Data at 0.3 mJ/cm^2 400-nm pulsed excitation. The first row shows the TA fit of the ground state bleach (GSB). *5HG obscured by pump.

λ_0 & HO	τ_0 and τ_1 (ps)	τ_2 Bi. Rec. (ps)	Amplitude
TA,GSB	IRF, 0.53±0.05	5.2±0.3	-
2320 nm THG	IRF	2.2±0.6	0.85
2320 nm 5HG	IRF	2.2±0.2	0.6
2050 nm THG*	IRF	3.5±0.3	0.75
1850 nm THG	0.21±0.02	5.7±5.1	0.6
1850 nm 5HG	0.14	3.2±0.2	0.6
1700 nm THG	0.26±0.01	-	0.85
1700 nm 5HG	0.13±0.03	-	0.92
1635 nm THG	0.41±0.06*	1.4±0.5	0.7
1550 nm THG	IRF	5.2±0.3	0.25
1500 nm THG	IRF	5.5±0.3	0.55
1440 nm THG	0.2±0.1	4.9±0.3	0.75

Table S3: Overview of extracted center frequency parameters at 0.3 mJ/cm^2 400-nm pulsed excitation. * The initial change is a redshift for τ_0, caused by the cross-phase modulation, afterwards a blueshift of +1 meV is observed until t=+1.5 ps. ** A redshift occurs after the initial EII blueshift.

λ_0 & HO	τ_0 and τ_1 (ps)	τ_2 Bi. rec. (ps)	Magnitude (meV)
2320 nm THG	IRF	3.9±0.7	+2
2320 nm 5HG	IRF	3.9±1.9	+10
2050 nm THG	IRF	3.6±0.3	+1*
1850 nm THG	0.68±0.05	2.6±0.2	+3
1850 nm 5HG	IRF	1.2	+5
1700 nm THG	0.43±0.02	3.9±1.4	+2
1700 nm 5HG	0.28±0.08	-	-1
1635 nm THG	IRF,0.45±0.9	0.48±0.1	-1.5
1550 nm THG	0.8±23	6.8±0.7	+1**
1500 nm THG	0.63±0.02	6.3±7.9	+2.5**
1440 nm THG	0.8±0.8, 1.1±1.1	4.7±0.7	+2, +1.4**

Some additional information can be gleaned from the FWHM behavior in table S4 which

Table S4: Complete Gaussian FWHM Data at 0.3 mJ/cm^2 400-nm pulsed excitation. * A rapid oscillation is observed in the first 1.5 ps. ** Initial broadening follows IRF, broadening peaks at 1 ps *** Change resembles noise around 0

λ_0 & HO	τ_0 and τ_1 (ps)	τ_2 Bi. rec. (ps)	Magnitude (meV)
2320 nm THG	IRF	1.7±0.2	-2
2320 nm 5HG	IRF	5.0±0.9	-8.5, -0.5
2050 nm THG**	IRF	3.6±0.3	+4
1850 nm THG	0.47±0.02	3.1±0.2	+12
1850 nm 5HG	0.40±0.04	1.5	-8
1700 nm THG	0.31±0.01	-	+6
1700 nm 5HG***	-	-	±0
1635 nm THG	IRF, 0.44±0.23	5.0±1.7	-2.5, -1.5
1550 nm THG	0.24±0.9	2.5±1.0	+4.5
1500 nm THG	0.27±0.03	4.0±28	+0.5
1440 nm THG	IRF, 0.29±0.07	3.2±0.7	+1

cannot be gleaned from the $\Delta\mu$ or amplitude changes. In certain data-sets, such as the third and fifth harmonics of 2320 nm in Fig.3A and B, the FWHM shows a rapid oscillation around pump-probe overlap. This is due to cross-phase modulation of the probe pulse as a result of the interaction of the pump pulse on the refractive index, causing rapid changes as a function of pump-probe delay during the temporal overlap.[18]

Theoretical Modeling

In this section, the parameters and assumptions of the EII and semiconductor Bloch equations are presented and discussed.

Semiconductor Bloch Equations

The behavior of the amplitude drop is theoretically explained through the semiconductor Bloch equations (SBE's) in a three-level system for $MAPbBr_3$.[19] The $\lambda_0 = 1500$-nm field intensity was fixed to $2 \times 10^{11}\,\mathrm{W/cm^2}$ with a pulse duration of 20 fs, or $4\,\mathrm{mJ/cm^2}$. The assumptions in this SBE simulation apply to the minimum of the amplitude, that is, once non-equilibrium distributions have cooled through HCC. The dephasing time (T_2) was set to half an optical cycle ($T_0 = 5\,\mathrm{fs}$) for the initial calculation, thus $T_2 = 2.5\,\mathrm{fs}$, but was reduced to 1.25 fs and 0.5 fs to calculate the effect of a significantly reduced T_2. The VB and two lowest CBs along the R-Γ direction are used, as the steepest band gradients of the VBs and CBs are situated along this crystal direction in the Brillouin zone.[20] The SBEs then calculate, in k-space, a system of coupled differential equations that use periodic boundaries matching the Brillouin zones of the crystal. This yields the interband polarization $P(\omega)$ and intraband current $J(\omega)$. The absolute square $|\omega^2 P(\omega) + i\omega J(\omega)|^2$ then yields the spectral density I_{HHG}, which is depicted in Fig. 4E.

Electron Impact Ionization

The center frequency shift is explained through an EII mechanism which occurs at high carrier densities. The justification is as follows: the 400-nm pump pulse excites carriers through single-photon excitation (SPE) into the conduction bands, creating an electron-hole plasma. This pump-induced electron-hole plasma acts as a seed population for EII driven by the IR probe laser pulse. Electron impact ionization is relatively efficient in the NIR spectral region due to the EII cross section being proportional to the wavelength (i.e. the

IR wavelength in this case) squared.[21] This is also why no center frequency shift is observed before pump-probe pulses, despite both pulses interacting with the material - the IR does not excite a significant carrier population to the conduction bands. The population transfer due to IR-driven EII induces a negative index of refraction change, resulting in an ionization induced blueshift of the IR. The EII becomes less efficient when fewer carriers are in the conduction bands, so the decrease in electron-hole plasma density through recombination mechanisms results in a decreasing blueshift.

The parameters for the simulation were chosen as follows: Both pulses are described by a Gaussian envelope. The 1440-nm IR probe duration at FWHM is taken to be 70 fs (based on the duration obtained through frequency-resolved optical gating (FROG) measurements). The 400-nm pulse duration at FWHM is taken to be 50 fs, which is an estimate based on the cross-correlation of the difference frequency generation of the known IR pulse duration and the unknown 400-nm pulse duration. The error on this estimate is around 10 fs, but does not affect the simulation significantly. The rate at which the pump creates a plasma through SPE is $W_{\text{SPE}} = \sigma * I_{\text{pump}}$, with σ the excitation cross-section and I_{pump} the intensity of the pump. As the pump pulse has a Gaussian shape, the resulting total plasma density ρ resembles an error function as a function of time. The effective electron mass m_{eff} is taken to be 0.2m_e, as experimental ranges are reported between 0.13 and 0.25m_e.[12,13,22] A lower effective electron mass would result in higher EII rates that are not observed. As the EII acts on the fundamental, the blueshift is simply multiplied by three for the third harmonic to match it.

The EII cross section σ_{EII} is described by:[21]

$$\sigma_{\text{EII}} = \frac{e^2}{n_{\text{be}}(\omega_0) m_{\text{eff}} c \epsilon_0} \frac{\tau_c}{1 + \omega_0^2 \tau_c^2} \tag{S6}$$

In which τ_c represents the characteristic collision time, ω_0 the angular frequency of the light, $n_{\text{be}}(\omega_0)$ the wavelength-dependent refractive index due to the bound electrons, ϵ_0 the

permittivity of free space, e the electron charge and c the speed of light. The relaxation parameter τ_{relax} was set to 1 ps. Excited electrons decay to lower conduction bands according to $W_{\text{Rel}} = \exp(-t/\tau_{\text{relax}})$, which in this approximation also accounts for any electron trapping. The real decay is better approximated by a bi-exponential decay. The impact rate is described by $W_{\text{EII}} = \alpha * \rho(t) * I_{\text{probe}}(t)$. Where $\alpha = \sigma_{\text{IMP}}/E_{\text{crit}}$ was found to be 6 2e−2 by fitting to the $\Delta(\mu)$ shift of the third harmonic of 1440 nm, as parameters are challenging to measure directly due to difficulty in accessing them. α is therefore numerically approximated and evaluated to see if it is indeed reasonable.

The total plasma density evolution $\frac{\partial \rho(t)}{\partial t}$ is dependent on the single-photon excitation rate W_{SPE}, the rate due to EII W_{EII} and the relaxation processes captured by W_{Rel} is described by:

$$\frac{\partial \rho(t)}{\partial t} = W_{\text{SPE}} + W_{\text{EII}} - W_{\text{Rel}} \tag{S6}$$

The ionization-induced blueshift rate along material depth $L = 300$ nm is then described by

$$\Delta\omega = \frac{e^2 L}{2c\epsilon_0 m_{\text{eff}}\omega} \frac{\partial \rho(t)}{\partial t} \tag{S6}$$

The blueshift resulting from the EII is then calculated for every timestep. The temporal profile of the THG blueshift is then gated by the normalized temporal profile of the IR probe. The result is a theoretical curve that agrees very well to the experimental data when simulated for the third harmonic of 1440 nm.

References

(1) Van Der Geest, M. L. S.; Mcgovern, L.; Vliet, S. V.; Zwaan, H. Y.; Grimaldi, G.; Boer, J. D.; Bliem, R.; Ehrler, B.; Kraus, P. M. Extreme-Ultraviolet Excited Scintillation of Methylammonium Lead Bromide Perovskites. *J. Phys. Chem. C* **2022**, *126*, 12554–12562.

(2) Van Der Geest, M. L. S.; Sadegh, N.; Meerwijk, T. M.; Wooning, E. I.; Wu, L.; Bloem, E.; Castellanos Ortega, S.; Brouwer, A. M.; Kraus, P. M. Extreme Ultraviolet-Excited Time-Resolved Luminescence Spectroscopy Using an Ultrafast Table-Top High-Harmonic Generation Source. *Rev. Sci. Instrum.* **2021**, *92*, 113004.

(3) Birowosuto, M. D.; Cortecchia, D.; Drozdowski, W.; Brylew, K.; Lachmanski, W.; Bruno, A.; Soci, C. X-ray scintillation in lead halide perovskite crystals. *Sci. Rep.* **2016**, *6*, 37254.

(4) Zürch, M.; Chang, H. T.; Borja, L. J.; Kraus, P. M.; Cushing, S. K.; Gandman, A.; Kaplan, C. J.; Oh, M. H.; Prell, J. S.; Prendergast, D.; Pemmaraju, C. D.; Neumark, D. M.; Leone, S. R. Direct and simultaneous observation of ultrafast electron and hole dynamics in germanium. *Nat. Commun.* **2017**, *8*, 15734.

(5) Ishteev, A. et al. Investigation of structural and optical properties of MAPbBr3 monocrystals under fast electron irradiation. *J. Mater. Chem. C* **2022**, *10*, 5821–5828.

(6) Mooney, J.; Kambhampati, P. Get the basics right: Jacobian conversion of wavelength and energy scales for quantitative analysis of emission spectra. *J. Phys. Chem. Lett.* **2013**, *4*, 3316–3318.

(7) Kojima, K.; Ikemura, K.; Matsumori, K.; Yamada, Y.; Kanemitsu, Y.; Chichibu, S. F. Internal quantum efficiency of radiation in a bulk $CH_3NH_3PbBr_3$ perovskite crystal quantified by using the omnidirectional photoluminescence spectroscopy. *APL Mater.* **2019**, *7*, 071116.

(8) Paradiz Dominguez, M.; Demirkurt, B.; Grzelka, M.; Bonn, D.; Galmiche, L.; Audebert, P.; Brouwer, A. M. Fluorescent liquid tetrazines. *Molecules* **2021**, *26*.

(9) Kamat, P. V.; Dimitrijevic, N. M.; Nozik, A. J. Dynamic Burstein-Moss shift in semiconductor colloids. *J. Phys. Chem.* **1989**, *93*, 2873–2875.

(10) Ghosh, S.; Ray, R.; Pal, S. K. Ultrafast Many-Particle Phenomena in Lead Bromide Hybrid Perovskite Nanocrystals under Strong Optical Excitation. *J. Phys. Chem. C* **2021**, *125*, 3198–3205.

(11) Baranowski, M.; Plochocka, P. Excitons in Metal-Halide Perovskites. *Adv. Energy Mater.* **2020**, *10*, 1903659.

(12) Baranowski, M. et al. Giant Fine Structure Splitting of the Bright Exciton in a Bulk MAPbBr3 Single Crystal. *Nano Lett.* **2019**, *19*, 7054–7061.

(13) Galkowski, K.; Mitioglu, A.; Miyata, A.; Plochocka, P.; Portugall, O.; Eperon, G. E.; Wang, J. T. W.; Stergiopoulos, T.; Stranks, S. D.; Snaith, H. J.; Nicholas, R. J. Determination of the exciton binding energy and effective masses for methylammonium and formamidinium lead tri-halide perovskite semiconductors. *Energy Environ. Sci.* **2016**, *9*, 962–970.

(14) Ščajev, P.; Miasojedovas, S.; Juršėnas, S. A carrier density dependent diffusion coefficient, recombination rate and diffusion length in $MAPbI_3$ and $MAPbBr_3$ crystals measured under one- And two-photon excitations. *J. Mater. Chem. C* **2020**, *8*, 10290–10301.

(15) Chan, C. C.; Fan, K.; Wang, H.; Huang, Z.; Novko, D.; Yan, K.; Xu, J.; Choy, W. C.; Lončarić, I.; Wong, K. S. Uncovering the Electron-Phonon Interplay and Dynamical Energy-Dissipation Mechanisms of Hot Carriers in Hybrid Lead Halide Perovskites. *Adv. Energy Mater.* **2021**, *11*, 2003071.

(16) Ashoka, A.; Tamming, R. R.; Girija, A. V.; Bretscher, H.; Verma, S. D.; Yang, S. D.; Lu, C. H.; Hodgkiss, J. M.; Ritchie, D.; Chen, C.; Smith, C. G.; Schnedermann, C.; Price, M. B.; Chen, K.; Rao, A. Extracting quantitative dielectric properties from pump-probe spectroscopy. *Nat. Commun.* **2022**, *13*, 1437.

(17) Park, J. S.; Choi, S.; Yan, Y.; Yang, Y.; Luther, J. M.; Wei, S. H.; Parilla, P.; Zhu, K. Electronic Structure and Optical Properties of α-$CH_3NH_3PbBr_3$ Perovskite Single Crystal. *J. Phys. Chem. Lett.* **2015**, *6*, 4304–4308.

(18) Dietzek, B.; Pascher, T.; Sundström, V.; Yartsev, A. Appearance of coherent artifact signals in femtosecond transient absorption spectroscopy in dependence on detector design. *Laser Phys. Lett.* **2007**, *4*, 38–43.

(19) Lindberg, M.; Koch, S. W. Effective Bloch equations for semiconductors. *Phys. Rev. B* **1988**, *38*, 3342–3350.

(20) Choudhary, S.; Shukla, A.; Chaudhary, J.; Verma, A. S. Extensive investigation of structural, electronic, optical, and thermoelectric properties of hybrid perovskite ($CH_3NH_3PbBr_3$) with mechanical stability constants. *Int. J. Energy Res.* **2020**, *44*, 11614–11628.

(21) Gulley, J. R.; Winkler, S. W.; Dennis, W. M.; Liebig, C. M.; Stoian, R. Interaction of ultrashort-laser pulses with induced undercritical plasmas in fused silica. *Phys. Rev. A - At. Mol. Opt. Phys.* **2012**, *85*, 013808.

(22) Zheng, K.; Zhu, Q.; Abdellah, M.; Messing, M. E.; Zhang, W.; Generalov, A.; Niu, Y.; Ribaud, L.; Canton, S. E.; Pullerits, T. Exciton Binding Energy and the Nature of Emissive States in Organometal Halide Perovskites. *J. Phys. Chem. Lett.* **2015**, *6*, 2969–2975.
