# Supplementary material for: Transient High-Harmonic Spectroscopy in an Inorganic–Organic Lead Halide Perovskite
Source: J Phys Chem Lett. 2023 Nov 28;14(48):10810–8. doi: 10.1021/acs.jpclett.3c02588 (PMC10711791; doi:10.1021/acs.jpclett.3c02588)
Supplement: Supplementary file 2 — jz3c02588_si_002.pdf [file jz3c02588_si_002.pdf]

jz-2023-025889.R1

Name: Peer Review Information for "Transient High-Harmonic Spectroscopy of an Inorganic-Organic Lead Halide Perovskite"

First Round of Reviewer Comments

Reviewer: 1

Comments to the Author

The manuscript "Transient High-Harmonic Spectroscopy of an Inorganic-Organic Lead Halide Perovskite" presents a study of high harmonic generation processes to monitor excited state dynamics in perovskite semiconductors. From the spectroscopy point of view, the work is very interesting, offering new insights into the nonlinear properties of these materials and the impact of photogenerated charge carriers, and merits publication. However, I believe the implications of the findings should be made clear to broaden the appeal of the manuscript. What information is found from transient SHHG that transient absorption could not reveal? If the work is presented as a demonstration of the method, what is the potential for future investigations?

Further to that, I have a few comments and questions:

- The photoexcitation fluence (400 nm) for the experiment is extremely high. How stable is the sample under those conditions? Is the fluence dependence shown in Fig S5 reflecting some saturation of absorption and/or photodegradation?

- Initially my main concern was the effect of refractive index changes and how it could influence the dynamics of the harmonic generation. The authors acknowledge these effects and discuss their impact on the frequency peak shift of the harmonics. The SI says the effect is negligible on the amplitude. Have the authors made a quantitative analysis of this? The high order nonlinear processes should be very strongly impacted by any small variation. On a similar note, I would expect the self-absorption of the harmonics that fall above the bandgap energy would have a significant impact and would change with dynamics of state filling. So it seems difficult to quantitatively separate all of these effects for a precise interpretation.

- Why does the population/depletion of electronic states impact the harmonic generation when the process is non-resonant? Do electric fields and screening effects associated with the presence of free charge carriers play a role?

- The authors mention some fast changes in the harmonics that are resonant with the exciton transition, and that this reflects exciton dissociation. The presence of free charges also bleaches the exciton transition in transient absorption, so how exactly do the harmonics generation is reacting differently to exciton or free charge populations?

A clarification of the points above would improve the appeal of the manuscript. I would also suggest a more clear organisation of the data and discussion, maybe separating the comparison of different types of measurements (i.e. 3HG vs 5HG vs 7HG, resonant vs non-resonant, etc) to improve readability.

Reviewer: 2

Comments to the Author

Authors explore high-harmonic generation (SHHG) spectroscopy to probe carrier cooling and relaxation in semiconductor materials, and compare their findings to well-known methods to probe these processes, such as transient absorption (TA). The halide perovskite methylammonium lead bromide (MAPbBr3) is used as a showcase. The work will be of interest to the community. Since the SHHG technique is not so well known, I think the manuscript will benefit from some clarifications, as listed below. In addition, the TA measurements at a single, high intensity give limited insight in recombination rate constants, especially for 1st and 2nd order processes that typically dominate at much lower intensities. I therefore advice to consider the following points in a revised version of the manuscript.

- What do the authors mean with “single-photon excitation” on page 3 line 31?

- Can the authors explain the concept of the SHHG method and measurements in more detail, maybe add some schemes?

- To what carrier densities (in /cm3) do the GW/cm2 intensities correspond, both in figure 1 and in the text?

- Can the authors comment on sample stability under the extremely high excitation densities (10^19/cm3)? How did they avoid melting/degradation of the MAPbBr3 perovskite thin film?

- Did the authors measure TA at lower intensities, to confirm the rate constants for uni- and bimolecular recombination? It seems likely that higher (3rd) order recombination processes will dominate under the conditions presented in the manuscript.

- What is the advantage of using SHHG instead of TA spectroscopy to study ultrafast carrier kinetics?

- The TA is used to understand photophysical processes and interpret the SHHG. Could one extract the same information from purely SHHG (i.e. without complementary techniques)?

- UV-VIS spectra of MAPbBr3 are not shown in the SI, while the technique is mentioned as analysis method.

Author's Response to Peer Review Comments:

ACS Publications
1155 Sixteenth Street N.W.
Washington, DC 20036

Ref.: **Response letter to reviewers' comments for Manuscript ID jz-2023-025889**

Amsterdam, October 19th, 2023

E-mail: kraus@arcnl.nl

Dear Prof.,
Dear Referees:

We express our gratitude to the reviewers for their expert feedback and valuable suggestions on the manuscript titled "Transient High-Harmonic Spectroscopy of an Inorganic-Organic Lead Halide Perovskite," authored by Maarten van der Geest *et al.* We sincerely acknowledge the reviewers' professional comments and constructive contributions, which have greatly enhanced the quality of our work. In this response letter, we present a comprehensive point-by-point response to each referee's comments. The referees' comments are highlighted in **blue**, while our responses are provided in **black**, and text added by us to the manuscript is **red**.

Report of Reviewer 1:

The manuscript "Transient High-Harmonic Spectroscopy of an Inorganic-Organic Lead Halide Perovskite" presents a study of high harmonic generation processes to monitor excited state dynamics in perovskite semiconductors. From the spectroscopy point of view, the work is very interesting, offering new insights into the nonlinear properties of these materials and the impact of photogenerated charge carriers, and merits publication.
We appreciate that the reviewer recognizes the relevance of our work.

1. However, I believe the implications of the findings should be made clear to broaden the appeal of the manuscript. What information is found from transient SHHG that transient absorption could not reveal? If the work is presented as a demonstration of the method, what is the potential for future investigations?
We thank the referee for encouraging us to elaborate more on the implications of our findings. We added the following sentences to the conclusion that highlight the potential of transient band structure measurements (page 16):
"The transient SHHG signal reduction, as observed in our study, can be rationalized by the increase in carrier density. Near-complete (Fig. S5) femtosecond signal extinction adds a new way of optical control: This capability has significant implications for future research and applications in ultrafast optics, as it opens the door to manipulating and controlling harmonic emission with unprecedented precision and speed. This has implications not only in fundamental research but also in practical applications such as ultrafast optoelectronic devices."
And the following sentence was added to the introduction (page 3):
"Combined with the previous insight that HHG is driven by laser-induced currents in bands[31], which led to all-optical band-structure reconstruction, our results pave the way for time-dependent band structure measurements. This capability is particularly relevant for materials where excitation induces drastic changes in the band structure, such as phase transitions in strongly correlated materials. "

2. The photoexcitation fluence (400 nm) for the experiment is extremely high. How stable is the sample under those conditions? Is the fluence dependence shown in Fig S5 reflecting some saturation of absorption and/or photodegradation?
Measurements were performed for several hours without significant sample degradation, the luminescence of the materials did not noticeably degrade.
The effects shown in Fig. S5 are a transient phenomenon, i.e. they are fully. The reduction in HHG emission strength is due to accelerated carrier dephasing as explained in Fig. 4. Saturation occurs because suppression reaches a maximum, i.e. due to very fast dephasing,
We added to the SI (p. S11): "The transient saturation in Fig. S5 is fully reversible. Absence of photodegradation was verified by monitoring and verifying the stability of luminescence from the samples."

3. Initially my main concern was the effect of refractive index changes and how it could influence the dynamics of the harmonic generation. The authors acknowledge these effects and discuss their impact on the frequency peak shift of the harmonics. The SI says the effect is negligible on the amplitude. Have the authors made a quantitative analysis of this? The high order nonlinear processes should be very strongly impacted by any small variation. On a similar note, I would expect the self-absorption of the harmonics that fall above the bandgap energy would have a significant impact and would change with dynamics of state filling. So it seems difficult to quantitatively separate all of these effects for a precise interpretation.
We thank the referee to address the important effects of refractive index changes on the HHG signal, which we have spent considerable effort to elucidate. We actually think the possibility to separate the HHG signal, to either reveal refractive index changes (via center frequency shift) or state blocking (via amplitudes) is one of the biggest strengths of the technique.
While the real part of the refractive index n is mainly responsible for phase matching due to different group velocities of fundamental and harmonics, the imaginary part of the refractive index k (i.e. the absorptive part) is responsible for reabsorption.
The sample thickness is 300 nm, which is less than the shortest emitted wavelengths, making phase matching effects and thus the real part n negligible.
We now focus on the imaginary part. The transient absorption measurements (Fig. 2) provide a direct estimate of the possible reabsorption of generated harmonics, and thus of the effect of changing absorption coefficients. Converting the optical densities in Fig. 2 to transmission changes, we find maximum effects on the amplitude of an up to 20% increase of transmission right at the band gap at 530 nm (2.3 eV), and a maximum decrease of up to 5% between 600-700 nm (1.65 eV – 2.25 eV). This cannot explain the observed signal changes, for the strongest suppression of H3 at the band gap around 530 nm, the corresponding absorbance change actually suggests an increased transmission, i.e. it has the opposite sign. This illustrates that the harmonics behave differently than the refractive index changes, which is caused by the microscopic HHG response, whereas the macroscopic phase matching and reabsorption is negligible due to the thin sample thickness.
We added the following to the manuscript (p. 15): "We note that the HHG amplitude changes are generally much stronger than the TA signals (Fig. 2) which suggests up to 20% increase of transmission right at the band gap at 530 nm (2.3 eV), and a maximum decrease of up to 5% between 600-700 nm (1.65 eV – 2.25 eV). This suggests that the HHG amplitude is not strongly affected by the changes of the complex-valued refractive index. Also changes in the real part should not affect the HHG amplitude, due to the low sample thickness, below the wavelength of the emitted light. However, the refractive index changes are captured in the center frequency shifts and broadening of the harmonics. As such, measuring center frequency shifts and amplitude changes in transient SHHG simultaneously provides and elegant way of tracing both state blocking and dephasing changes through amplitude, and refractive index changes through center frequencies."

4. Why does the population/depletion of electronic states impact the harmonic generation when the process is non-resonant? Do electric fields and screening effects associated with the presence of free charge carriers play a role?
We thank the referee for encouraging us to make this point clearer.
Excitation increases electron scattering which decreases the dephasing time T_2, which reduces the HHG signal. This is elaborated in the context of Fig. 4, pages 12-13, as well as ref. 49.
We also added "Simultaneously, a pre-excited carrier distribution has an effect on electron scattering and thus on electron-hole dephasing, as discussed in Fig. 4." on page 9 of the manuscript to emphasize the effect more clearly.

5. The authors mention some fast changes in the harmonics that are resonant with the exciton transition, and that this reflects exciton dissociation. The presence of free charges also bleaches the exciton transition in transient absorption, so how exactly do the harmonics generation is reacting differently to exciton or free charge populations?
Generally, HHG is sensitive to resonances and strongly enhances those. In this particular case, it is likely that the exciton enhances both multi-photon excitation, as the exciton is three-photon resonant, as well as the radiative interband polarization, often thought of as electron-hole recollision (added to p. 16). Notably, Fig. 3C clearly shows a transient enhancement feature at the exciton resonance which is absent in the TA measurements (Fig. 2). This is likely explained because (i) the HHG measurements have a better time resolution (HHG is confined to below the pulse duration of the driver, whereas white-light generation needed for TA generally stretches the pulses), and (ii) the HHG measurements are more sensitive due to the reasons stated above, whereas exciton dissociation in TA strongly overlaps with ground-state bleaching.

6. A clarification of the points above would improve the appeal of the manuscript. I would also suggest a more clear organisation of the data and discussion, maybe separating the comparison of different types of measurements (i.e. 3HG vs 5HG vs 7HG, resonant vs non-resonant, etc) to improve readability.
We thank the referee for the suggestion to improve the general appeal, which we addressed by providing a new figure (Fig. 3), as outlined in the comment 2 to referee 2 below.

Report of Reviewer 2

Authors explore high-harmonic generation (SHHG) spectroscopy to probe carrier cooling and relaxation in semiconductor materials, and compare their findings to well-known methods to probe these processes, such as transient absorption (TA). The halide perovskite methylammonium lead bromide (MAPbBr3) is used as a showcase. The work will be of interest to the community. Since the SHHG technique is not so well known, I think the manuscript will benefit from some clarifications, as listed below. In addition, the TA measurements at a single, high intensity give limited insight in recombination rate constants, especially for 1st and 2nd order processes that typically dominate at much lower intensities. I therefore advice to consider the following points in a revised version of the manuscript.

1. What do the authors mean with “single-photon excitation” on page 3 line 31?
We thank the referee suggesting a clarification: We use the term “single-photon excitation”, i.e. exciting carriers from the valence to conduction bands with a single photon, to stress the difference with multi-photon excitation. We added “single-photon excitation (i.e. we overcome the band gap with one photon)” which we added to the text on page 3.

2. Can the authors explain the concept of the SHHG method and measurements in more detail, maybe add some schemes?
We thank the referee for helping to make our manuscript more appealing. We provide an extra figure (Fig. 3) with some extra schemes to illustrate the concept of the SHHG method.

3. To what carrier densities (in /cm3) do the GW/cm2 intensities correspond, both in figure 1 and in the text?
Carrier densities are not a good measure for the intensity of the fundamental in SHHG experiments. As the fundamental driver is far below band gap, there is not much excitation by the fundamental. Moreover, as we are looking at non-linear processes, the pulse duration, in addition to energy per area, is an important parameter Thus, to avoid confusion, we stick with the convention of the community and report the fundamental intensity, while we report fluences and carrier densities for the pump pulses that directly (by a one-photon transition) excite the band gap.

4. Can the authors comment on sample stability under the extremely high excitation densities (10^19/cm3)? How did they avoid melting/degradation of the MAPbBr3 perovskite thin film?
We thank the referee for pointing us to the importance of possible photodegradation. We replied to the effects of photodegradation in detail in comment 2 to referee 1. In short, no melting/degradation was observed.

5. Did the authors measure TA at lower intensities, to confirm the rate constants for uni- and bimolecular recombination? It seems likely that higher (3rd) order recombination processes will dominate under the conditions presented in the manuscript.

The rate constant we measured are fully consistent with pervious results in literature (refs. [17, 33, 34]), and were also confirmed by independent measurements at lower intensities.
Below we include a TA spectrum recorded at a pump-intensity of 1.2±0.6*10^{18} cm^{-3}. The value of τ_2 is within the error bars of the τ_2 value at 2.2*10^{19} cm^{-3}, as is the value of τ_1. Extracted values in both cases are longer than the temporal resolution of the TA setup (approximately 0.2 ps).

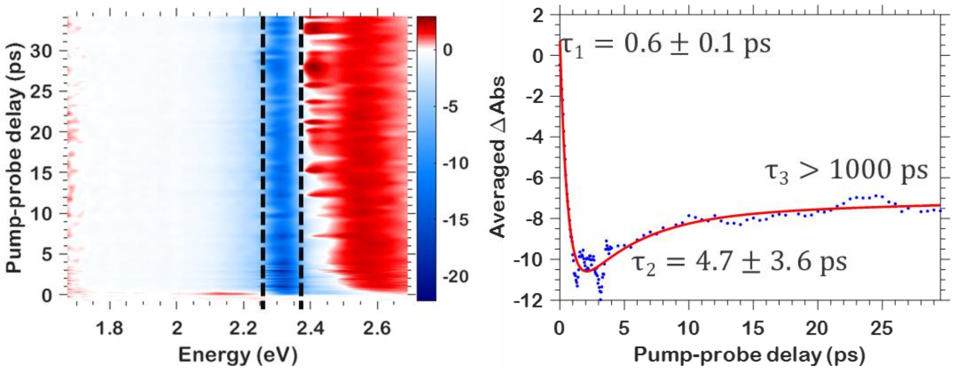

6. What is the advantage of using SHHG instead of TA spectroscopy to study ultrafast carrier kinetics?

We added the mentioned advantages to both introduction and conclusion, see comment 1 of referee 1.

7. The TA is used to understand photophysical processes and interpret the SHHG. Could one extract the same information from purely SHHG (i.e. without complementary techniques)?

The referee points towards an important strength of SHHG. Indeed, all photophysical primary processes and time scales can in principle be independently obtained from SHHG, but more confirmation of our results would be needed. As an example, excited carrier populations map directly to refractive index changes and thus frequency shifts, so tracing the center frequencies follows recombination rates of states at the emitted energy. This of course needs to be tested more in the future. We added on p. 15: "As such, measuring center frequency shifts and amplitude changes in transient SHHG simultaneously provides and elegant way of tracing both state blocking and dephasing changes through amplitude, and refractive index changes through center frequencies. The sensitivity to state blocking via frequency sifts of harmonics may allow measurement of wavelength-resolved primary processes such as recombination rates, similar to transient absorption."

8. UV-VIS spectra of MAPbBr3 are not shown in the SI, while the technique is mentioned as analysis method.

The UV-VIS spectra are shown in Fig. 1, together with the harmonic emission. We changed the text in Fig.1 and p.4 slightly to A) UV-Vis absorption spectrum of the \ce{MAPbBr3} thin film sample under normal incidence (red, right y-axis)…. and The ultraviolet-visible (UV-Vis) absorption spectrum of the \ce{MAPbBr3} sample under normal incidence is depicted… respectively.

We again thank the referees for their thorough work and look forward to a final assessment.

On behalf of the authors:
Sincerely yours,

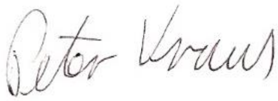

Peter Kraus,
Group leader of High-harmonic generation and EUV Science
Assistant professor of Physics at Vrije Universiteit Amsterdam
